# Sm_26.25_Ge_22.75_O_5_: Oxidic Sm_30_Ge_4_O_5_ Superclusters Embedded in a Zintl Polyanionic Framework

**DOI:** 10.1021/acs.inorgchem.6c00775

**Published:** 2026-04-07

**Authors:** Joju Sabu Mathew, Vitaliy Romaka, Ulrich Burkhardt, Thomas Doert, Julia-Maria Hübner

**Affiliations:** a Faculty of Chemistry and Food Chemistry, 98896 TUD Dresden University of Technology, 01062 Dresden, Germany; b Max Planck Institute for Chemical Physics of Solids, Nöthnitzer Straße. 40, 01187 Dresden, Germany

## Abstract

The heteroanionic rare-earth germanide oxide Sm_26.25_Ge_22.75_O_5_ was obtained by arc melting. The crystal structure determined by single-crystal X-ray diffraction (space group *P*4/*nmm*, *a* = 14.9838(2), *c* = 10.5353(1) Å) features [Sm_6_O] octahedra and capped [Sm_8_Ge] prisms that are condensed to Sm_30_Ge_4_O_5_ superclusters. These clusters form an interpenetrating framework with polyanionic Ge chains. Chemical bonding analysis reveals the substantial role of oxygen in stabilizing the structure: in the hypothetical oxygen-free compound, significant electron localization appears at the centers of Sm_6_ octahedra, coinciding with the experimentally observed oxygen positions. Consistently, DFT calculations show that the incorporation of oxygen lowers the enthalpy of formation in comparison to the hypothetical intermetallic compound. Electronic structure calculations indicate metallic conductivity for both the suboxide and the hypothetical oxygen-free intermetallic compound.

## Graphical abstract

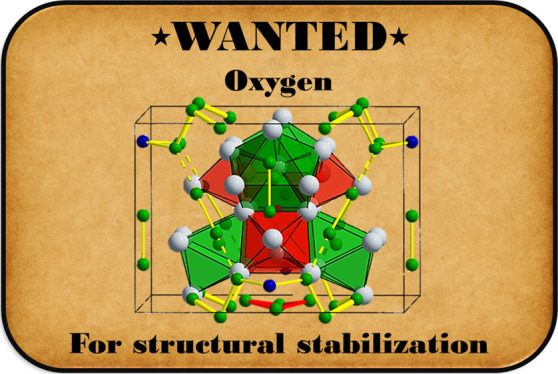

## 1 Introduction

Intermetallic compounds exhibit a wide range of structural and electronic properties that arise from different degrees of electron transfer and bonding.^1^ Rather than forming sharply separated classes, these compounds span a continuum of bonding motifs, from electron-deficient metallic frameworks to electron-precise polyanionic networks.^2^ The Zintl–Klemm concept describes one important limiting case within this landscape, in which formal electron transfer leads to valence-satisfied (poly-)­anionic substructures of compounds with semiconducting behavior that can often be rationalized using classical two-center–two-electron bonding schemes.^3,4^

However, many compounds formally classified as Zintl phases deviate from this idealized picture. Metallic conductivity is frequently observed despite formal valence balance, reflecting incomplete electron localization^5^ and the presence of multicenter interactions.^6^ Prominent examples include Ba_3_Si_4_,^7^ CaSi_2_,^8,9^ and CaSi,^10,11^ which exhibit metallic behavior despite precise electron counts. These observations highlight that the Zintl–Klemm concept is best regarded as a structural guideline rather than a strict predictor of electronic properties.

Zintl anions are highly basic, chemically reactive, and particularly susceptible to oxidation, opening up diverse reaction pathways. This tendency is further enhanced in electron-rich Zintl phases by the presence of “excess” electrons, which has important chemical consequences.^12^ The prevailing reactivity has been exploited synthetically to access unusual allotropes and metastable compounds.^13,14^ In contrast, the pathway of oxidation of Zintl phases usually proceeds to completion, resulting in the breakdown of the polyanionic framework and the formation of thermodynamically stable, salt-like oxides. In order to prevent the complete structural collapse and to isolate intermediates along this oxidation pathway, specific kinetic windows have to be hit. From a thermodynamic perspective, such partially oxidized, so-called suboxides are significantly less stable than fully oxidized products. As a consequence, their formation is restricted to narrow kinetic windows, often at elevated temperatures, and isolation in phase-pure form is challenging. From this kinetic perspective, these intermetallic suboxides may be considered as metastable phases, at least at ambient conditions.

At low *p*-block element content, such partial oxidation typically results in the formation of classical oxide-centered motifs stabilizing salt-like structures, as observed in *A*_9_ *M*O_4_ (*A* = Rb, Cs; *M* = Al, Ga, In, Fe, Sc),^15,16^ as well as in Ca_3_AsN^17^ and Mg_5_Bi_3_H_*x*_.^18^ For intermediate *p*-block element contents, one option is the formation of additional, discrete *p*-block clusters^19^ as exemplified by Tl_8_ and Tl_6_ units in Cs_8_Tl_8_O^20^ and *A*_10_Tl_6_O_2_ (*A* = K, Rb).^21^ In contrast, a small and chemically distinct class of compounds exhibits a remarkably different response to oxidation. In these so-called oxypnictides,^22–26^ or pnictide halides,^27,28^ oxidations proceeds in a localized and highly ordered manner by selectively stabilizing nonmetal atom-filled metal clusters by reducing the density of states at the Fermi energy that provides a significant stabilization to the electronic structure, while preserving extended Zintl polyanionic frameworks, such as in La_26_Ge_19_ *M*_5_O_5_ (*M* = Ag, Cu).^29^ The coexistence of metal clusters and polyanionic frameworks results in multiple bonding environments and underlines the structural and electronic distinctiveness of these compounds.

Herein, a new member of the group of heteroanionic compounds, Sm_26.25_Ge_22.75_O_5_, is presented, which was observed during the investigation of phase relations in the Sm–Ge–O system.

## 2 Experimental Section

### 2.1 Synthesis

Sm_26.25_Ge_22.75_O_5_ crystals were first found as a byproduct in the synthesis of SmGe. For that, samarium (Chempur, rod, 99.90% metal basis) and germanium (Alfa Aesar, pieces >2 mm, 99,999%) were weighed with a nominal composition of Sm_62.5_Ge_37.5_ (Sm excess was used to compensate for loss through evaporation during synthesis). The powders were mixed and pressed to a pellet. Subsequently, the pellet was molten in an arc melter located in an argon-filled glovebox three times, and the sample was turned in between to increase homogeneity. Please note that oxygen was contained as an impurity in the rare earth metal despite its stated, high purity.

After confirmation of the composition, two other targeted routes were applied. First, pellet-pressed samples of Sm, Ge and GeO_2_ or Sm_2_O_3_ (weighed in the stoichiometric ratio of the target compound Sm_26.25_Ge_22.75_O_5_ plus up to 10% Sm excess) were arc melted. However, the samples immediately separated upon arc melting into a black, nanocrystalline powder and silver-colored ingots. This finding is consistent with previous studies,^30^ so this synthesis route was not deemed promising and, therefore, not pursued further. Pressed pellets of Sm and Ge (26.25:22.75 plus 10% Sm excess to compensate for evaporation loss) were also molten in an arc melter located in ambient atmosphere (MAM-1, Edmund Bühler). This arc melter was only partially evacuated until 300 mbar and filled with Ar up to 600 mbar to generate an atmosphere consisting of air and Ar. The sample was turned and remolten three times to increase homogeneity.

Second, samples of Sm, Ge and GeO_2_ or Sm_2_O_3_ (weighed in the stoichiometric ratio of the target compound Sm_26.25_Ge_22.75_O_5_ and with Sm excess of up to 10%) were mixed, pressed and sealed in evaporated glass ampules, both with and without using glassy carbon or tantalum crucibles. The samples were heated in muffle furnaces at 800 or 900 °C for 99 h followed by cooling within 5 h or by quenching the ampule in water. According to powder X-ray diffraction measurements (PXRD), the target phase was not identified in these samples.

### 2.2 Powder X-Ray Diffraction (PXRD)

PXRD was performed in Bragg–Brentano geometry on an Empyrean diffractometer (PANalytical) equipped with a curved Ge(111)-monochromator using Cu-*Kα*_1_ radiation (λ = 1.5406 Å). For data analysis, the programs STOE WinXPow v.2.08 ^31^ and Jana2020 ^32^ were used.

### 2.3 Metallographic Analysis

Prior to analysis, samples were embedded and polished using disks with diamond powders (grain sizes 6, 3, and 0.25 μm). Energy-dispersive X-ray spectroscopy (EDXS) was conducted with a Jeol JSM 7800 F scanning electron microscope and an attached EDX detector (XFlash silicon drift detector, Bruker Nano, Berlin). Wavelength-dispersive X-ray spectroscopy (WDXS) was carried out with a Cameca SX100 electron microprobe equipped with a tungsten cathode. The WDX spectrometer was equipped with PET (pentaerythritol) or LiF monochromator crystals. As standard, samples of the compound *hp*-Sm_3_Ge_5_ were used.^33^ The X-ray emission lines Sm-*L*_α_ were excited at an electron beam of 20 keV and a beam current of 8 nA and for Ge-*K*_α_ at 20 keV and 30 nA.

The oxygen content was calculated based on the sum of the weight percentages of Sm and Ge that deviated from 100%.

### 2.4 Single Crystal X-Ray Diffraction (SXRD)

SXRD data were obtained on a Rigaku Synergy S diffractometer with Ag–Kα radiation (λ = 0.56087 Å) and equipped with a hybrid pixel array detector (Dectris Eiger2 1M) at 100 K. Data integration and scaling were performed using CrysAlisPro v.42.49.^34^ Numerical absorption correction was conducted based on a Gaussian integration over a multifaceted crystal model. The structure was solved using Superflip,^35^ and refined in Jana2020.^32^ Remeasuring the crystal after 6 months of storage in air showed no signs of structural decomposition. Deposition Number 2495545 contains the supplementary crystallographic data for this paper. These data are provided free of charge by the joint Cambridge Crystallographic Data Centre and Fachinformationszentrum Karlsruhe Access Structures service. For visualization, POV-Ray (version 3.7) was used.

### 2.5 Quantum Chemical Calculations

DFT based calculations were carried out using the Vienna Ab-initio Simulation Package (VASP) v.6.5.1 ^36,37^ with the projector augmented wave (PAW) method and the Perdew–Burke–Ernzerhof (PBE) exchange-correlation functional within the generalized gradient approximation (GGA).^38^ A plane-wave cutoff energy of 500 eV was used. The Brillouin zone was sampled using a Monkhorst–Pack^39^ *k*-point mesh of 11 × 11 × 16, corresponding to a *k*-point spacing of 0.006 Å^–1^. Input files for band structure calculations were generated with VASPKIT.^40^ The following PAW potentials with the corresponding valence electron configurations were used: Sm (5*s* ^2^ 5*p* ^6^ 5*d* ^1^ 6*s* ^2^), Ge (3*d* ^10^ 4*s* ^2^ 4*p* ^2^), and O (2*s* ^2^ 2*p* ^4^). In the case of Sm, the potential corresponds to the Sm^3+^ ion with the *f*-electrons kept frozen in the core. The structures, comprising an ordered arrangement solely accounting for the disorder on position Ge1 by introducing 50% Ge and 50% Sm on this position and the subsequent construction of an ordered arrangement in space group *P*1 both for the suboxide Sm_26.5_Ge_22.5_O_5_ and the hypothetical compound Sm_26.5_Ge_22.5_, were fully relaxed prior to all subsequent electronic structure calculations, with atomic positions and cell parameters optimized until the forces on each atom were below the convergence threshold. The elastic constants were calculated based on the energy-strain method implemented in VASPKIT.

Population analyses were conducted based on the wave function projections from the VASP calculations using the LOBSTER code (version 5.1.1).^41^ The Bader analyses were performed using the Bader code^42^ (version 1.05) to evaluate charge distribution and bonding characteristics. For visualization, VESTA was used.^43^

## 3 Results & Discussion

### 3.1 Phase Relations

During arc melting experiments of samples with a nominal composition Sm_62.5_Ge_37.5_ (to compensate for mass loss during synthesis), a majority phase corresponding to an unknown compound was discovered. The unknown phase could be indexed with a structure similar to that reported for Er_26_Ge_22.77_ ^44^ (space group *P*4/*nmm*) with lattice parameters *a* = 15.0234(4), *c* = 10.5657(5) Å (Figure S1), though the existence of Er_26_Ge_22.77_ as a distinct phase remains uncertain and may also require stabilization by oxygen (see discussion below). The Sm:Ge ratio determined by WDXS corresponds to Sm_51.3_Ge_48.7_ (Sm_25.1_Ge_23.9_) (Figure S3, Table S1), which is in fair agreement with the composition Sm_26.25_Ge_22.75_O_5_ determined from a single crystal refinement (see below). The oxygen content was estimated as the difference of the sum of Sm and Ge to 100% owing to the well-known difficulties in the quantification of light elements by WDXS.^45,46^ Importantly, no evidence for the presence of other light elements, such as nitrogen, was found in these measurements.

Based on the established composition of the new phase, different reaction routes were tested for a targeted synthesis of a phase pure sample. Solid state reactions starting from Sm and Ge, with or without addition of either Sm_2_O_3_ or GeO_2_ at temperatures up to 900 °C for several days, followed by either quenching in water or slow cooling, did not yield the suboxide. Arc melting using Sm from a different supplier likewise did not yield the target phase, leading to the conclusion that oxygen originates from impurities present in the elemental samarium. In contrast, arc melting under a mixed air-argon atmosphere resulted in the formation of the target phase (Figure S2); however, no air-to-argon ratio was identified that produced phase-pure samples. This behavior, along with the microstructure (see below), suggests that the target compound is most likely a high-temperature phase and that the cooling rate achievable in common synthesis routes is insufficient to ensure complete homogenization during solidification. Consistently, Er_26_Ge_22.77_,^44^ and the structurally related compounds Ho_26_Pd_4_(Pd,Ge)_19‑x_ ^47^ and La_26_Ge_19_ *M*_5_O_5_ (*M* = Ag, Cu),^29^ are described as high-temperature phases obtained by quenching as well.

The microstructure of an arc-melted sample exhibits distinct regions (Figure 1a) indicating formation under markedly different solidification conditions. This heterogeneity is readily explained by the strong thermal gradients inherent to arc melting, where the outer regions of the melt droplet solidify rapidly, while the central volume remains at elevated temperature for at least several seconds. The right-hand region is dominated by a fine-grained microstructure composed primarily of the novel oxide Sm_26.25_Ge_22.75_O_5_, identified based on the Sm:Ge ratio by EDXS, together with SmGe. Sm_26.25_Ge_22.75_O_5_ crystallizes as the primary phase and exhibits predominantly columnar to platelet-like morphologies. The sharp edges and well-developed crystallites indicate formation under rapid solidification conditions, where insufficient time was available to reduce interfacial energy by interface rounding. Notably, no binary oxide is observed in this region, supporting the interpretation that Sm_26.25_Ge_22.75_O_5_ forms directly from the melt rather than by postsolidification oxidation. On the left, right at the border of both regions (Figure 1b), a pronounced droplet-like morphology of the highest-melting binary phase Sm_5_Ge_3_ is observed,^48^ accompanied by segregated binary samarium oxide (see elemental distribution maps in Figure S4). With increasing distance from the interface, inclusions of a more germanium-rich phase, tentatively assigned to Sm_2_Ge_3_ or SmGe_1.8_, become more frequent (Figure 1a). This trend is consistent with progressive enrichment of germanium in the residual melt following the primary crystallization of Sm_5_Ge_3_. The absence of Sm_26.25_Ge_22.75_O_5_ after annealing at temperatures up to 900 °C can be rationalized on the basis of the metallographic observations, suggesting that this compound is a high-temperature phase formed by fast quenching and that slower cooling rates foster the segregation of SmO_*x*_ instead of the formation of the binary compound. Additional evidence for slow cooling in the left-hand region is provided by the presence of isolated pores within the microstructure, which likely originate from phase segregation accompanied by a reduction in specific volume. According to PXRD analysis, the sample consists of approximately 50 wt % Sm_26_Ge_23_O_5_, 30 wt % SmGe, 15 wt % Sm_3_Ge_5_, and 5 wt % Sm_5_Ge_3_, in good agreement with the phase distribution inferred from metallography. The binary samarium oxide is not detected by PXRD, consistent with its minor abundance and localized occurrence.

**Figure 1:**
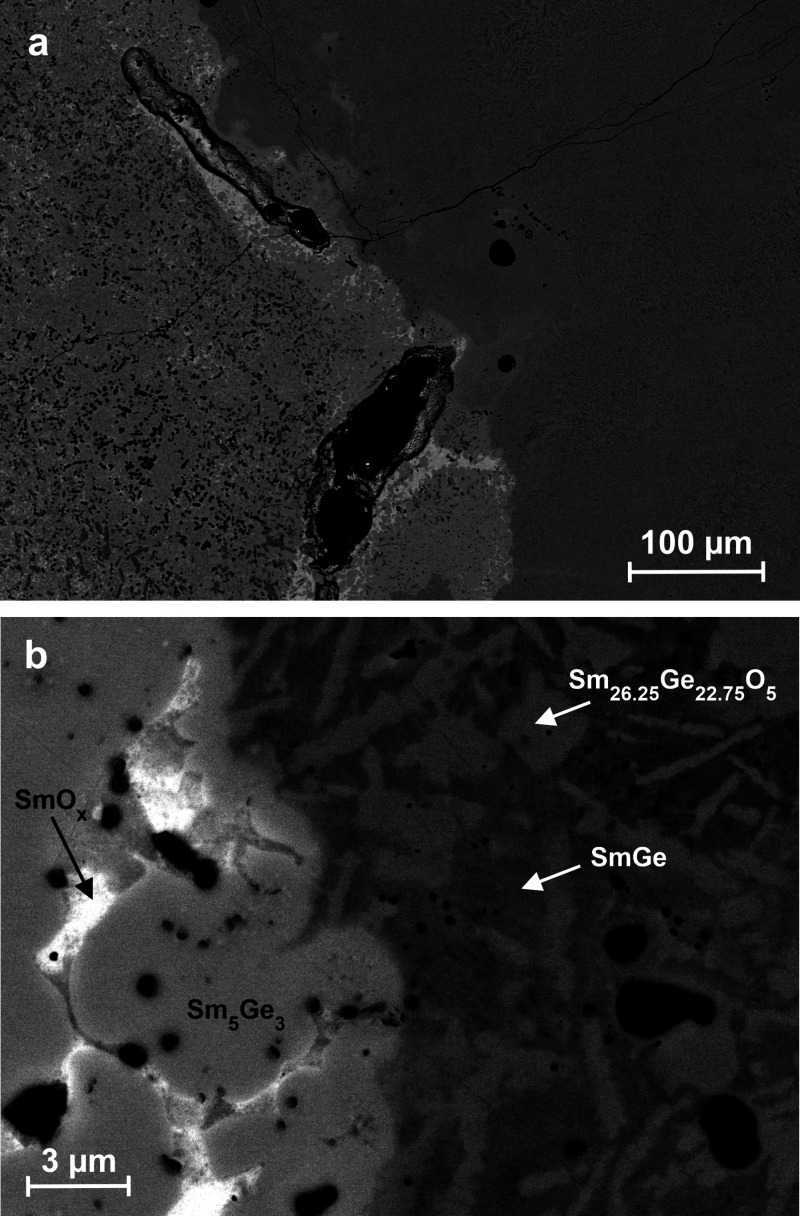
SEM image (BSE contrast) of a sample with nominal composition Sm_62.5_Ge_37.5_ (to compensate for mass loss during synthesis) after arc melting of Sm (containing SmO_*x*_) and α-Ge. (a) The specimen exhibits two clearly distinct regions formed under different cooling rates. (b) The rapidly cooled region (right) shows primary crystallites of Sm_26_Ge_23_O_5_. The slowly cooled region (left) is dominated by primary Sm_5_Ge_3_ together with segregated SmO_*x*_.

### 3.2 Crystal Structure Determination & Refinement

From carefully crushing the ingot obtained by arc melting, black crystals of the title compound were isolated. Single crystal diffraction experiments confirmed space group *P*4/*nmm* (*a* = 14.9838(2), *c* = 10.5353(1) Å) and no structural transition was observed upon cooling to 100 K. Structure solution^35^ revealed six samarium and eight germanium atomic positions, which are consistent with the structure reported for Er_26_Ge_22.77_.^44^ However, refinement within this structural model yielded residuals of *R*_1_ = 0.037 and *wR*_2_ = 0.114, along with residual electron densities of 10.99 *e* ^–^/Å^3^. These relatively high residual values were observed despite the low *R*_int_ value of 0.026 for the selected crystal, indicating its high quality. WDXS measurements revealed, alongside the expected Sm and Ge emission lines, an additional peak at the characteristic energy of oxygen, suggesting its incorporation within the sample. Residual electron density observed in the Fourier difference map could be satisfactorily modeled by the inclusion of oxygen atoms at the respective positions. Moreover, a significantly too high electron density around the Ge1 position and strongly prolate displacement parameters for Ge1, Ge2 and Ge6, were found. While the displacement anomalies could be resolved by introducing partially occupied split positions, the refined occupancies of Ge1 split sites resulted in a physically meaningless occupation of about 120%. As no other element apart from Sm, Ge and O were found in WDXS analysis, a partial occupation with Sm is the only explanation for the high electron density. In an alternative approach, this site (Ge1_2/Sm; Table S3) was, thus, modeled as being partially occupied by Sm (for further details on the modeled disorder, see Figure S6). A nonrestrained refinement resulted in a nearly full occupation of the site Ge1 with an approximate amount of 25% Sm on Ge1_2, consistent with the formula Sm_26.25_Ge_22.75_O_5_ (see Table S4). The final residuals are *R*_1_ = 0.0157 and *wR*_2_ = 0.0423 and the residual electron density is featureless –0.61/0.54 e/Å^3^ (Table S2). No ordered and chemically meaningful structure model could be achieved in subgroups of *P*4/*nmm.* Therefore, the presented model is deemed as the best fit to the data.

As the interatomic distance between Ge1_2/Sm and the closest neighbor Ge7 of about 2.73 Å is shorter than typical Sm–Ge distances (∼3 Å),^33,49–52^ the occupancy of Ge7 was refined. This yielded an occupancy of 99.6(3) %, which is not considered a significant reduction from 100%. Shifting Ge1_2/Sm away from the site 2*c* resulted in unstable refinements. DFT optimization of an ordered structure model of Sm_26.5_Ge_22.5_O_5_ (no split positions, 50% Sm and 50% Ge on Ge1, for further details, see below) revealed a larger and physically reasonable distance between Ge1/Sm and Ge7 of ∼2.85 Å which is close to the sum of their covalent radii, while the distance from Ge1_2 to Ge7 remained almost unchanged (∼2.74 Å). Therefore, the observed short Sm–Ge distance is probably an artifact of the refinement caused by domains of slightly different compositions and ordering patterns. The presence of Sm and Ge on the same crystallographic position may be the consequence of incomplete ordering during the quenching process, pointing again to Sm_26.25_Ge_22.75_O_5_ being a high-temperature phase.

### 3.3 Crystal Structure Description

The crystal structure of Sm_26.25_Ge_22.75_O_5_ (Figure 2, for atom labels see Figure S5) consists of four principle structural building blocks: Sm octahedra centered by O, capped trigonal prisms of Sm surrounding Ge, a three-dimensional Ge network and isolated Ge butterfly anions. One central [OSm_6_]-octahedron shares faces with four adjacent octahedra, forming a [O_5_Sm_18_]-cluster (Figure 3). The capped trigonal Sm-prisms around Sm share in turn faces with three surrounding [OSm_6_]-octahedra, forming a [Sm_30_Ge_4_O_5_] supercluster that is fully enclosing the central octahedron. The superclusters share corners with neighboring superclusters, resulting in a 3D net (Figure 2b).

**Figure 2:**
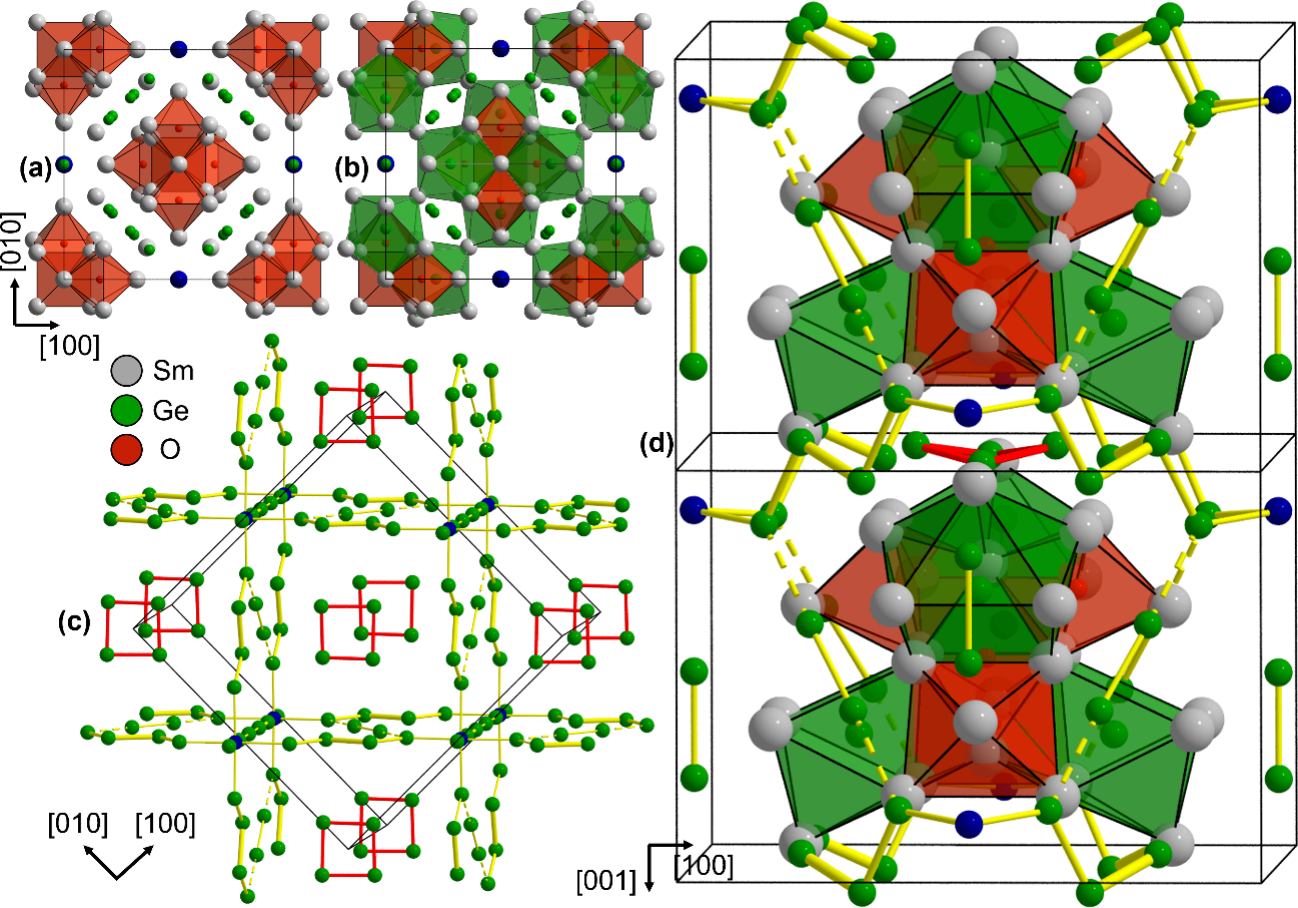
Crystal structure of Sm_26.25_Ge_22.75_O_5_. (a) O@Sm_6_ octahedra in red and (b) capped trigonal Sm-prisms surrounding Ge in green, forming a supercluster. (c) Polyanionic Ge–Ge substructure. The bond thickness scales with the bond lengths with short interatomic distances being represented by thicker lines, broken lines correspond to distances >3 Å. The dark blue sphere marks the partially occupied site entailing Sm. (d) Arrangements of clusters (red and green), polyanionic Ge-subnetwork (yellow) and isolated butterfly anions (red). For split positions, only highest occupied sites are shown for clarity.

**Figure 3:**
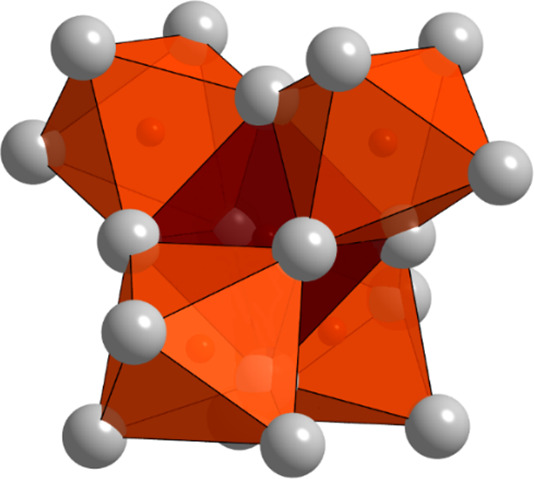
[O_5_Sm_18_]-cluster consisting of five face-sharing [OSm_6_]-octahedra.

Metal octahedra *M*_6_ *X* (*X* = O, N, C, B etc.) are a recurring structural motif in different cluster compounds, intermetallics, and some metal-rich oxides, nitrides, etc.^15,16,20,21,29,53–60^ These octahedra can occur as isolated clusters, share edges or faces to form columns or larger clusters, or link into extended networks (see Table S5).^29,60–64^ The [O_5_ *M*_18_]-cluster consisting of five face-sharing [O*M*_6_]-octahedra is also observed in Sr_21_Si_2_O_5_C_6_ ^65^ and Ba_21_ *Tt*_2_O_5_ (*Tt* = Si, Ge),^66^ redetermined as Ba_21_(*Tt*/Tr)_2_O_5_H_*x*_ (*Tt*/Tr = Ge, Si, Ga, In, Tl).^67^ In these, the [O_5_ *M*_18_]-cluster is further enclosed by tetrel- or triel-centered metal icosahedra. The same supercluster [*RE*_30_Ge_4_O_5_] (*RE* = rare earth metal) arrangement was only found to occur in La_26_Ge_19_ *M*_5_O_5_ (*M* = Ag, Cu),^29^ whereas for Er_26_Ge_22.77_ ^44^ and Ho_26_Pd_4_(Pd,Ge)_19‑x_,^47^ the same supercluster was reported without oxygen incorporation, raising the question of the existence of the oxygen-free intermetallic structure or an oversight of oxygen positions.

The supercluster is surrounded by a polyanionic Ge network, resulting in an interpenetrating 3D network of superclusters and Ge polyanionic framework (Figure 2d). The Ge network (Figure 2c) consists of finite Ge chains with distances *d*_Ge–Ge_ ranging between 2.50 and 2.59 Å, in turn connected to a 3D network via longer *d*_Ge–Ge_ of up to 2.73 Å (Table S4). These values fall within the range of similar Ge–Ge chains.^68^

Interestingly, all split positions (Ge1, Ge2 and Ge6) are arranged in a linear manner in the *bc*-plane (Figure S6). In La_26_Ge_19_ *M*_5_O_5_ (*M* = Ag, Cu),^30^ the Ag/Cu incorporation takes place at the site equivalent to position Ge1 in the title structure – the same position where we found a too high electron density – and similarly elongated displacement ellipsoids are observed in this linear string of atoms. In La_26_Ge_19_ *M*_5_O_5_ (*M* = Ag, Cu),^30^ another Ag/Cu atom is located on the position equivalent to Ge7 in Sm_26.25_Ge_22.75_O_5_, giving rise to a strikingly short Ag–Ag distance of about 2.6 Å.

Stacked along [001], isolated Ge_4_ butterfly anions separate the supercluster in *c*-direction (red in Figure 2). In contrast to the folded geometry of butterfly anions in *rt*-Ba_3_Ge_4_ ^69^ (opening angle ca. 110°), the ones in Sm_26.25_Ge_22.75_O_5_ adopt an almost planar arrangement with an enlarged angle of ca. 150°. The peripheral Ge–Ge distances (2.56 Å) closely match those in *rt*-Ba_3_Ge_4_, whereas the latter exhibits an additional short diagonal contact (2.58 Å), resulting in a butterfly consisting of two edge-sharing triangles. The corresponding diagonal in the present compound is significantly longer (3.58 Å), thus resulting in an almost square geometry of the Ge_4_ unit.

For the Sm_30_Ge_4_O_5_ supercluster, the total number of vertices is 39 (30 Sm, 4 Ge, 5 O). Applying Wade’s and Jemmis’ rules for polyhedral clusters, the skeletal electron requirement for this closo-type cluster is 80 electrons. Considering contributions from the metal and *p*-block atoms in the cluster (30 Sm × 3 *e* ^–^, 4 Ge × 4 *e* ^–^, 5 O × 2 *e* ^–^), the total electron requirement for the two superclusters per unit cell is 160 *e* ^–^, while Sm, Ge, and O provide 232 *e* ^–^, leaving 72 residual electrons. The Zintl polyanionic network requires 68 electrons (calculated based on connectivity and Wyckoff positions, see Table S3s and S4), giving a formal electron excess of ∼4 *e* ^–^. While the simple electron-counting analysis provides a useful guideline for understanding the supercluster and polyanionic Ge network, a more rigorous investigation of the chemical bonding has been performed using DFT calculations.

To explore how these distinct structural units can exist side by side in this structure, electronic structure and chemical bonding analyses were performed. The calculated Bader, Mulliken, and Löwdin atomic charges (Table S6) reveal a clear cationic nature of the Sm atoms and the anionic character of Ge and O, consistent with the difference in their electronegativities.

In the O@Sm_6_ octahedra (Table S7), the -IpCOHP of the Sm–O pairs (Figure S7) ranges between 1.78 and 2.54 eV, which is typical for metal oxides. The corresponding ICOBI values of 0.28–0.42 indicate a significant ionic contribution to the bonding, while the value 2 × ICOBI represents the number of shared electrons between two atoms and is interpreted to reflect the electron-deficient multicenter bonding. In momentum space, the bonding (-pCOHP and pCOBI) features two distinct maxima in the range of –4 to –6 eV caused by the interaction between the *p*-states of O and the *sd*-states of Sm. The corresponding band structure shows distinct, mostly flat Sm and O bands in that energy range (Figure S13). Between –4 eV and the Fermi level, the occupied *d*-states of Sm contribute negligibly to the bonding, while the empty *d*-states of Sm above the Fermi level exhibit a distinct antibonding character. The ELF distribution (Figure 4) reveals electron localization at the O^2–^ anion, and spherical localization around Sm.

**Figure 4:**
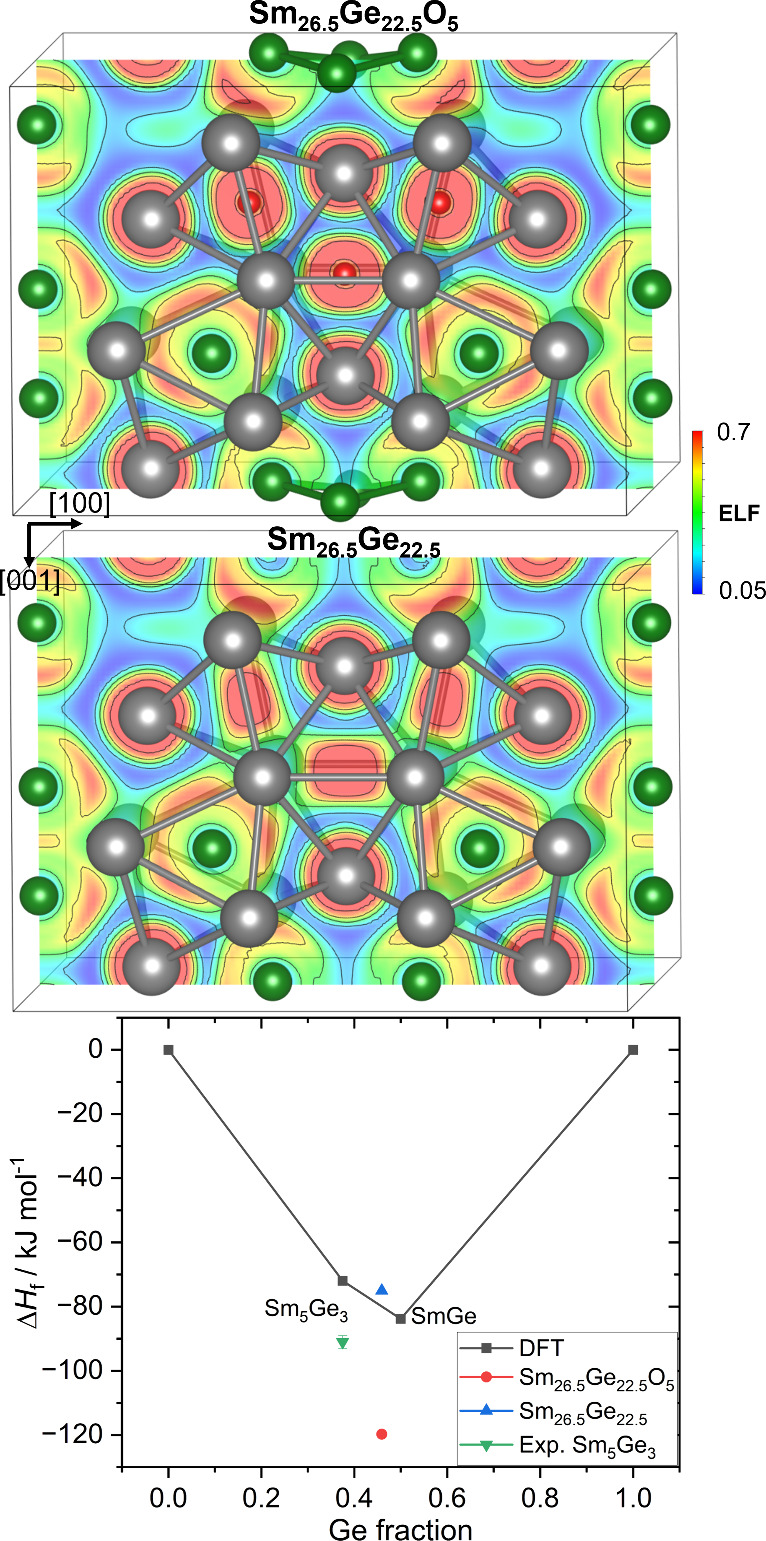
Distribution of the ELF for the optimized (top) suboxide Sm_26.5_Ge_22.5_O_5_ and the hypothetical (middle) Sm_26.25_Ge_22.75_. Convex hull of the Sm–Ge system (bottom) in the concentration region around the hypothetical Sm_26.5_Ge_22.5_ compound.

The bonding between Sm and Ge within the overcapped trigonal prisms of Sm around Ge (Ge@Sm polyhedra) is characterized by purely bonding states below the Fermi level (Figure S8), and is antibonding above it. The interaction of the *sd*-states of Sm and the *s*-states of Ge results in bonding at –8 eV. In the range from –4 eV to the Fermi level, the *sd*-states of Sm overlap with the *p*-states of Ge. While the -IpCOHP values (Table S7) lie in the range of 1.24 to 2.27 eV and the corresponding ICOBI between 0.27 and 0.47, depending on the Sm–Ge interatomic distance, the overall bonding picture is similar across all Sm–Ge bonds. The 2 × ICOBI values also indicate an electron-deficient, multicenter Sm–Ge bonding. The ELF is concentrated around more electronegative Ge, indicating partial ionic character.

The Ge-butterflies represent a closed-chain of Ge–Ge bonding caused by mixing of the *s* and *p* states of Ge in the range from –6 to –11 eV (Figure S9). At –4 eV, the *p*-states of Ge are mainly bonding, while above this energy their bonding character gradually decreases and becomes antibonding at –1 eV, which indicates their partial electron lone pair (ELP) character. The -IpCOHP of 3.11 eV and IpCOBI of 0.56 (Table S7) are significantly higher than those for the Sm–O and Sm–Ge interactions, while the amount of 1.11 shared electrons indicates a stronger interaction. The electron localization between Ge atoms is clearly observable as a maximum on the ELF plot, with additional strong electron localization on Ge atoms indicating the presence of ELPs.

The bonding between Ge atoms in the Ge-chains is similar to the one in Ge-butterflies; however, the Ge *s*-states are more delocalized, and the Ge8–Ge7 *p*-states mixing has stronger antibonding character than Ge8–Ge8 (Figure S10). The ELF around the Ge8 and Ge7 atoms has two well-established maxima (ELPs) with a minor difference on the Ge7 atom due to the proximity to the Sm/Ge1_2 position.

In the 3D Ge-network, the Ge4–Ge4 interactions show the weakest contribution from *s*-*p* mixing (Figure S11), making the *s* states solely responsible for the bonding states in the range of –6 to –9 eV. The *p*-states of Ge also exhibit a bonding character in the range of –2 to –4 eV. The states between –1 and –2 eV give a strongly antibonding effect, resulting in smaller -IpCOHP (2.56 eV) and IpCOBI (0.46) values (Table S7). The ELF around these Ge atoms has a circular shape, which could be assigned to some lone pair character, while between the atoms, a small localization is observed.

The bonding between Ge4 and Ge7 atoms, with the highest observable interatomic distance, shows the weakest contribution of the *s*-states mixing (−6 to –8 eV) to the bonding (Figure S11), while the *p*-interactions have a bonding character in the range (−1 to –4 eV). Above –1 eV, the *p*-states start to mix, acquiring an antibonding character, which further weakens the bonding.

To elucidate the formation mechanism of the Sm_26.5_Ge_22.5_O_5_ suboxide and to assess the feasibility of obtaining the oxygen-free intermetallic compound, the enthalpies of formation were calculated (Figure 4). The enthalpy of formation of Sm_26.5_Ge_22.5_O_5_ (−119.72 kJ·mol^–1^) was found to be considerably more negative than that of the hypothetical oxygen-free Sm_26.5_Ge_22.5_ compound (−75.05 kJ·mol^–1^), indicating a higher thermodynamic stability of the suboxide.

Additionally, DFT calculations were performed for two binary phases with higher and lower Ge contents relative to the hypothetical Sm_26.5_Ge_22.5_ composition—namely, Sm_5_Ge_3_ (Mn_5_Si_3_-type) and SmGe (CrB-type). The tie-line connecting Sm_5_Ge_3_ and SmGe of the convex hull lies below the enthalpy of Sm_26.5_Ge_22.5_, suggesting that the latter is thermodynamically unstable. In contrast, the incorporation of oxygen results in a more negative enthalpy of formation below the tie-line, thereby stabilizing the compound Sm_26.5_Ge_22.5_O_5_. Nevertheless, comparing the calculated formation enthalpies of the suboxide and the oxygen-free binary phases with experimental data of stable GeO_2_ and Sm_2_O_3_ oxides shows that the suboxide is slightly above the Sm_2_O_3_–GeO_2_–Sm_5_Ge_3_ tie-triangle (Figure S12), indicating its potential metastable origin. For the description of the bonding features of Sm_26.5_Ge_22.5_, see SI.

The distribution of the density of electronic states (DOS) for both compounds predict metallic conductivity (Figure S13) with the Fermi level located in the pseudogap for the suboxide.

The elastic properties were modeled to assess the mechanical stability of the compounds. The crystal structure of Sm_26.5_Ge_22.5_O_5_ and the hypothetical Sm_26.5_Ge_22.5_ compound belong to the high-symmetry tetragonal class with six independent elastic constants (*c*11, *c*12, *c*13, *c*33, *c*44, and *c*66) and no coupling between shear and normal strains (*c*16 = 0) (Table S8). In comparison to the intermetallic, the Sm_26.5_Ge_22.5_O_5_ suboxide exhibits higher values of *B*, *E*, and *G* (Table S9), indicating stronger bonding, higher stiffness and hardness, and thus lower compressibility. For both compounds, the Pugh’s ratio is below the 1.75 threshold, and thus classifying them as brittle. The Cauchy Pressure (*P*_c_ = *c*12 - *c*44) is negative in both cases, indicating dominant localized (directional) bonding character, which is more pronounced in Sm_26.5_Ge_22.5_O_5_. The Vickers hardness *VH* calculated from Chen’s and Tian’s models shows comparable values, which are higher for the suboxide and consistent with its higher *E* and *G* values. The Sm_26.5_Ge_22.5_O_5_ compound is characterized by a higher Debye temperature (299.1 K) compared to the hypothetical Sm_26.5_Ge_22.5_ (258.4 K), revealing its higher lattice stiffness and stronger covalent bonding.

All eigenvalues of the stiffness matrices are positive and satisfy the elastic stability criteria^70^ for both compounds. Both compounds are stiffer within the basal (*a*-*b*) plane (*c*11 > *c*33), reflecting stronger in-plane atomic interactions and weaker interlayer bonding, which is in agreement with the location and chemical bonding analysis of the Ge-butterflies and Ge-chains. The *c*12 and *c*13 show similar values in both cases, but *c*13 > *c*12, meaning stronger coupling between *a*(*b*) and *c* directions than between *a* and *b*. The *c*44 and *c*66, which are responsible for the resistance to shear on different planes, are significantly smaller than *c*11 and *c*33, indicating that shear deformation occurs much more easily than normal deformation and is typical of brittle crystalline materials.

## 4 Conclusions

In this work, the new heteroanionic framework compound Sm_26.25_Ge_22.75_O_5_ was synthesized. The structure features distinct, coexisting structural building blocks: [Sm_18_O_5_] clusters consisting of face-sharing [Sm_6_O] octahedra, which further share faces with four capped trigonal Sm-prisms centered by Ge. The resulting supercluster is embedded in a 3D polyanionic Ge network consisting of Ge-chains and Ge_4_ butterfly units. The close structural relation to La_26_Ge_19_ *M*_5_O_5_, Er_26_Ge_22.77_ and Ho_26_Pd_4_(Pd,Ge)_19‑x_, suggests that a broader family of related compounds could be synthesized. As DFT calculations indicate that the binary Sm_26.5_Ge_22.5_ is unstable relative to the suboxide, the related Ho and Er compounds may contain oxygen as well, making a structural redetermination worthwhile. Compared to other suboxides, Sm_26.25_Ge_22.75_O_5_ exhibits a more diverse bonding scenario: in addition to ionic, metallic, and cluster-type bonding, significant covalent interactions are also present. These unique bonding features and directional interactions may make this family of compounds relevant in contexts where both electronic and mechanical properties are important. Moreover, incorporation of different rare-earth metals could enable magnetic functionality.

## Supplementary Material



## Notes

Details on sample characterization, including powder X-ray diffraction, metallographic analysis (including energy- and wavelength-dispersive spectroscopy), and structural analysis by single crystal diffraction, as well as details on electronic structure and chemical bonding calculations (PDF)
