# Supplementary material for: Sm26.25Ge22.75O5: Oxidic Sm30Ge4O5 Superclusters Embedded in a Zintl Polyanionic Framework
Source: Inorg Chem. 2026 Apr 7;65(15):8201–9. doi: 10.1021/acs.inorgchem.6c00775 (PMC13100948; doi:10.1021/acs.inorgchem.6c00775)
Supplement: Supplementary file 1 [file ic6c00775_si_001.pdf]

SUPPORTING INFORMATION

$Sm_{26.25}Ge_{22.75}O_5$: Oxidic $Sm_{30}Ge_4O_5$ superclusters embedded in a Zintl polyanionic framework

Joju Sabu Mathew,[a] Vitaliy Romaka,[a] Ulrich Burkhardt,[b] Thomas Doert,[a] and Julia-Maria Hübner[a*]

[a] Faculty of Chemistry and Food Chemistry, TUD Dresden University of Technology, 01062 Dresden, Germany

[b] Max Planck Institute for Chemical Physics of Solids, Nöthnitzer Straße. 40, 01187 Dresden, Germany

* julia-maria.huebner@tu-dresden.de

1. Powder X-ray diffraction (PXRD)
2. Energy- and Wavelength-dispersive X-ray spectroscopy
3. Single crystal refinement
4. Structural motifs in related compounds
5. Electronic structure and chemical bonding

1. Powder X-ray diffraction (PXRD)

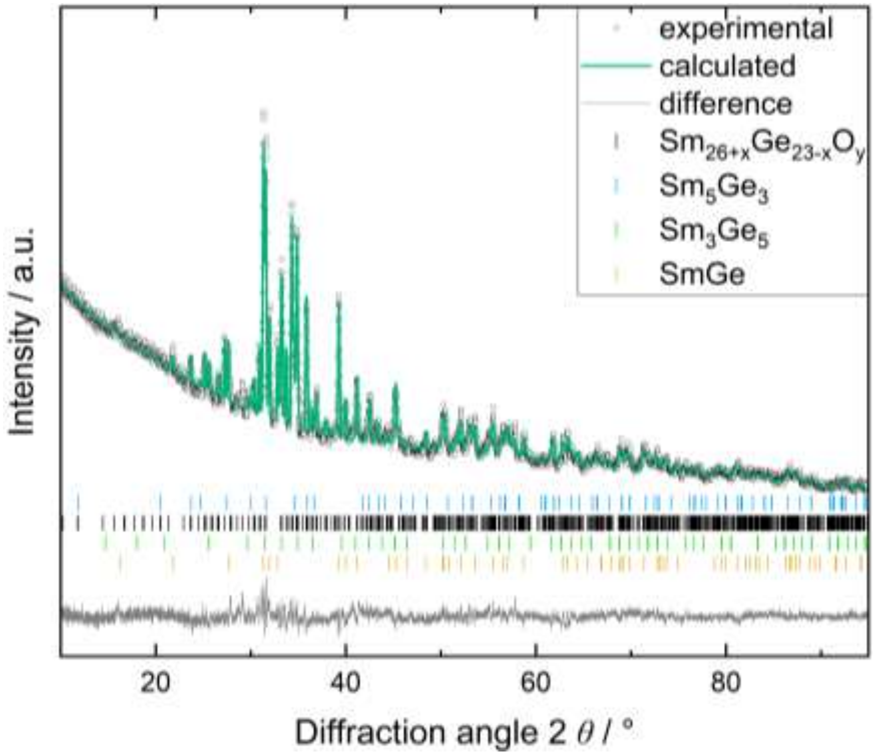

Figure S1. Rietveld refinement using diffraction data of a sample obtained by arc melting Sm and Ge in argon. The refinement resulted in 29 wt.-% SmGe, 14 wt.-% Sm_3Ge_5, 5 wt.-% Sm_5Ge_3 and 52 wt.-% $Sm_{26+x}Ge_{23-x}O_y$.

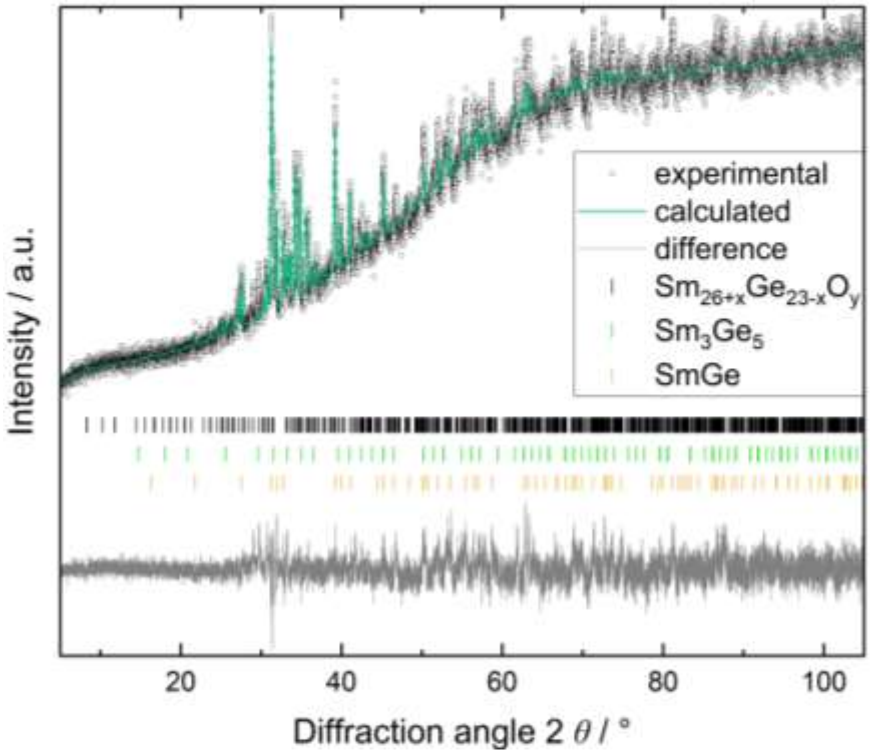

Figure S2. Rietveld refinement using diffraction data of a sample obtained by arc melting Sm and Ge in 300 mbar air and argon. The refinement resulted in 48 wt.-% SmGe, 7 wt.-% Sm_3Ge_5, and 45 wt.-% $Sm_{26+x}Ge_{23-x}O_y$. Additional peaks of an unknown side phase were observed, that did not match known binaries.

2. Energy- and Wavelength-dispersive X-ray spectroscopy

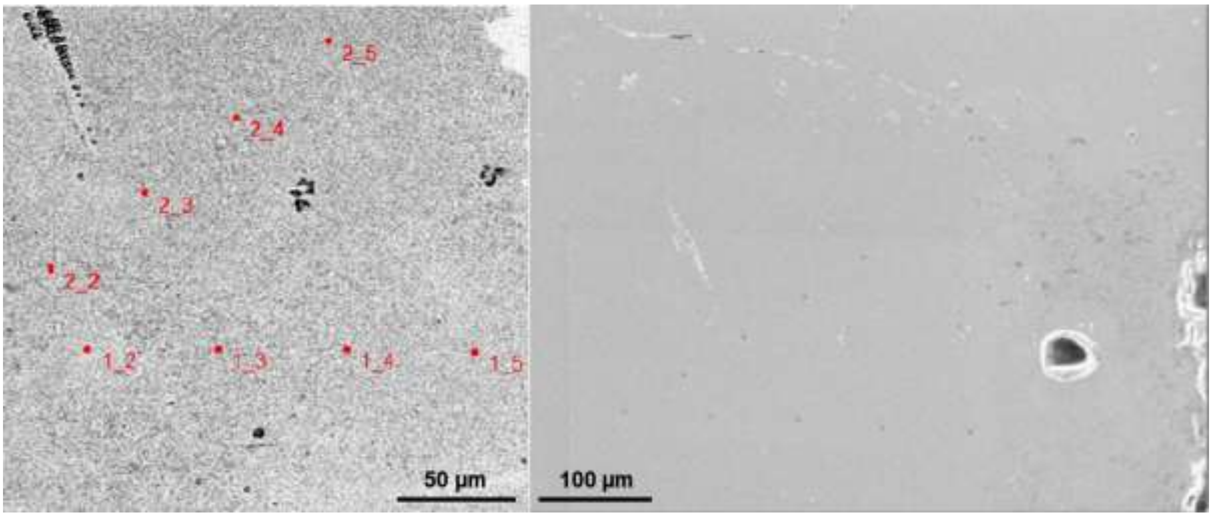

Figure S3. Microprobe images of a sample obtained by arc melting Sm and Ge in argon. (left) backscattered and (right) secondary electron image.

Table S1. Results of WDXS measurements. Points correspond to the positions marked in red in Figure S3. From the average, the formula $Sm_{26.5}Ge_{25.2}O_{2.4}$ or $Sm_{25.1}Ge_{23.9}$ (without O) would result, respectively.

#	Sm	Ge	O
1_2	53.1	46.9	0
1_3	44.6	44.4	11.0
1_4	47.4	44.3	8.3
1_5	51.6	48.4	0
2_2	45.5	45.1	9.4
2_3	48.9	48.4	2.7
2_4	52.2	47.2	0.6
2_5	48.9	48.0	3.1
**Average**	**49.0**	**46.6**	**4.4**
**Average without O**	**51.3**	**48.7**	

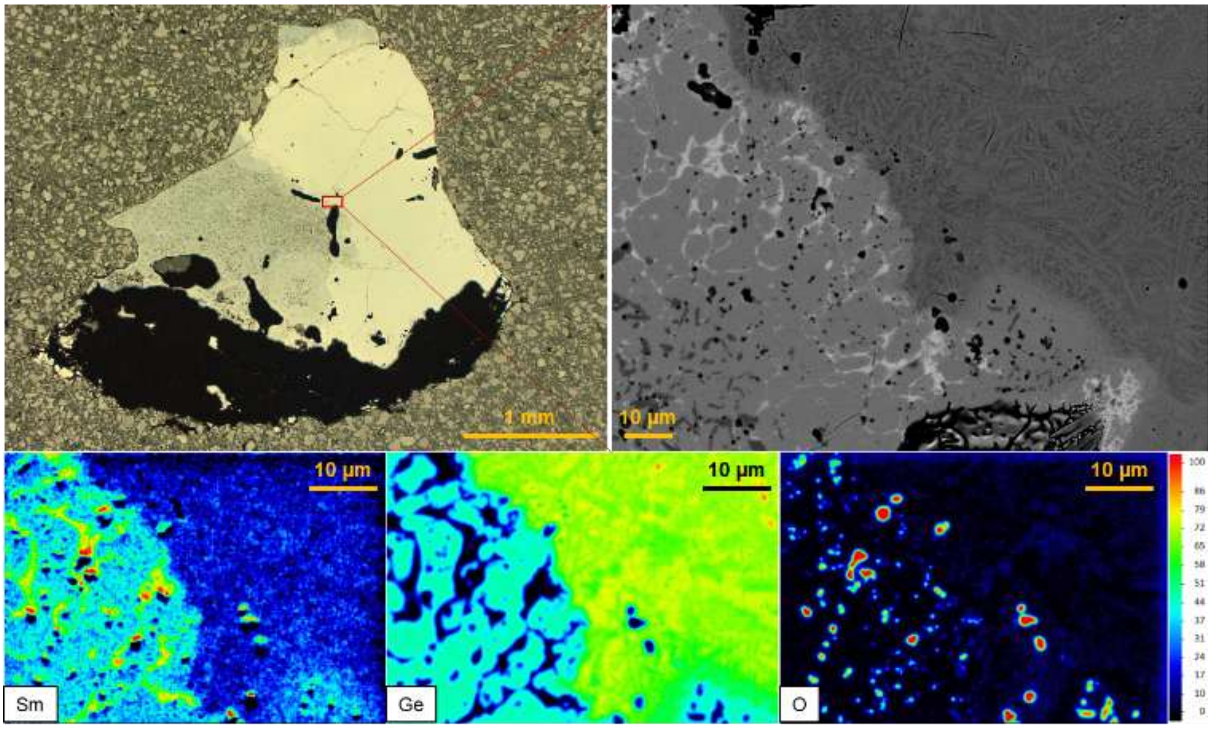

Figure S4. (top, left) Light microscopy (bright field) and (top, right) SEM (BSE) images of an arc melted sample. The element composition of the left part of the sample match Sm_5Ge_3, Sm_3Ge_5 and SmO_x, the right upper part to SmGe and $\sim Sm_{26}Ge_{23}O_x$ as determined by WDXS. (bottom) SEM/EDXS maps showing Sm, Ge and O distribution within the sample.

3. Single crystal refinement

Table S2. X-ray diffraction data for $Sm_{26.25}Ge_{22.75}O_5$. Further details on the crystal structure investigations can be obtained from the Fachinformationszentrum Karlsruhe (http://www.fiz-karlsruhe.de/request_for_deposited_data.html) on quoting the depository number 2495545.

Composition	$Sm_{26.25}Ge_{22.75}O_5$
Space group	*P*4/*nmm* (129, origin choice 1)
Unit cell parameters	
a [Å]	14.9838(2)
c [Å]	10.5353(1)
V [Å^3]	2365.33(5)
Formula units	2
Diffractometer, measurement temperature	XtaLAB Synergy, Dualflex, Eiger2 1M, Ag *K*α radiation, 100 K
Reflections collected/independent within *I*>3σ(I)	23900 / 3255
Refined parameters	91
Measurement range	$-25 \le h \le 23$, $-23 \le k \le 20$, $-17 \le l \le 14$
Fourier difference ρ_{min}/ρ_{max} [e$^-$/Å^3]	-0.61 / 0.54
Residuals / GoF	R_1=0.0157, wR_1=0.0405, R_2=0.0196, wR_2=0.0423 / 1.23

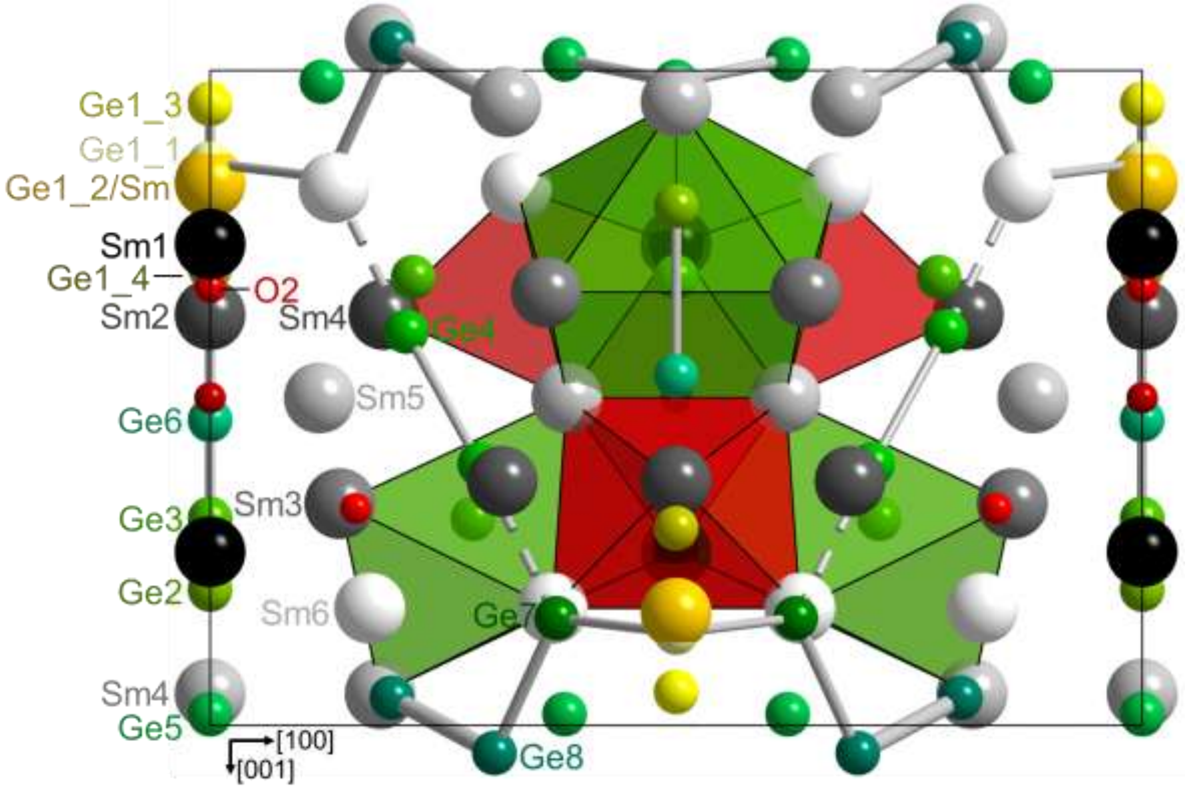

Figure S5. Crystal structure comprising atom labels.

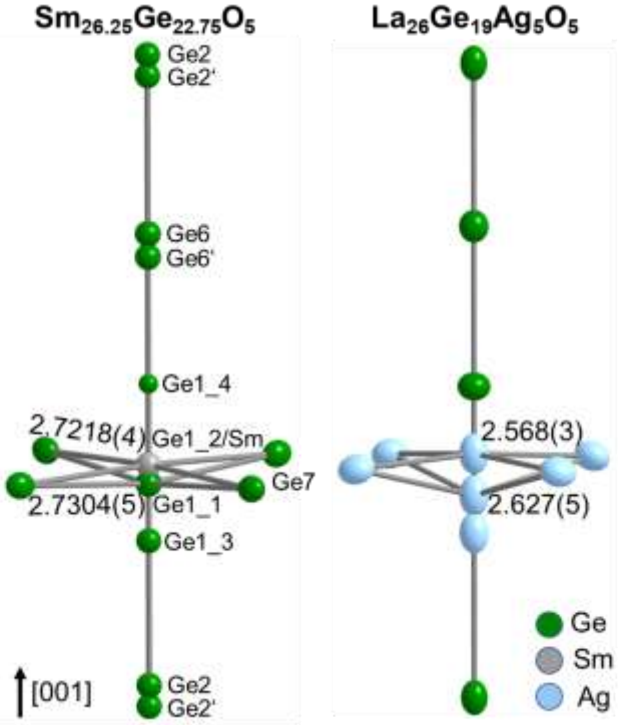

Figure S6. Linear arrangement of split positions Ge1, Ge2 and Ge6 in comparison to equivalent positions in $La_{26}Ge_{19}Ag_5O_5$.[1] Selected distances are given in Å. Ellipsoids are shown with a 90 % probability.

Table S3. Atomic positions, site occupancies, Wyckoff positions and displacement parameters for $Sm_{26.25}Ge_{22.75}O_5$.

Atom	Type	Occup.	Site	*a* / *x*	*b* / *y*	*c* / *z*	U_{ani}*/U_{iso}
Sm1	Sm	1	4*f*	½	½	0.73501(2)	0.00630(6)
Sm2	Sm	1	8*i*	½	0.81345(1)	0.62728(2)	0.00720(5)
Sm3	Sm	1	8*j*	0.36076(1)	*x*+½	0.33865(2)	0.00683(4)
Sm4	Sm	1	8*i*	½	0.81747(1)	0.04799(2)	0.00703(5)
Sm5	Sm	1	8*h*	0.382741(9)	$\bar{x}$	½	0.00665(4)
Sm6	Sm	1	16*k*	0.367197(9)	0.671323(9)	0.82110(1)	0.00680(3)
Ge1_1	Ge	0.67(2)	2*c*	0	½	0.1418(4)	0.0077(4)
Sm/Ge1_2	Sm	0.25(1)	2*c*	½	0	0.8280(6)	0.0072(7)
Ge1_3	Ge	0.038(5)	2*c*	0	½	0.051(2)	0.007(6)
Ge1_4	Ge	0.031(3)	2*c*	0	½	0.300(2)	0.0042
Ge2	Ge	0.172(9)	2*c*	½	0	0.178(1)	0.0072(2)
Ge2‘	Ge	0.828(9)	2*c*	½	0	0.2112(3)	0.0072(2)
Ge3	Ge	1	8*i*	½	0.71802(3)	0.31547(4)	0.00713(9)
Ge4	Ge	1	8*j*	0.28938(2)	*x*+½	0.60135(4)	0.00688(7)
Ge5	Ge	1	8*i*	½	0.61968(3)	0.01694(4)	0.0073(1)
Ge6	Ge	0.817(7)	2*c*	½	0	0.4622(2)	0.0075(2)
Ge6’	Ge	0.183(7)	2*c*	½	0	0.4987(9)	0.0075(2)
Ge7	Ge	1	8*j*	0.37162(2)	*x*+½	0.83609(4)	0.00853(9)
Ge8	Ge	1	8*j*	0.30505(2)	*x*+½	0.04221(4)	0.0071(1)
O1	O	1	2*b*	½	½	½	0.009(1)
O2	O	1	8*i*	0.3440(2)	½	0.3310(3)	0.0118(5)

*Sm1: U_{11}= 0.0065(1), U_{22}= 0.0062(1), U_{33}= 0.0063(1), U_{12}=U_{13}=U_{23}=0; Sm2: U_{11}= 0.00625(8), U_{22}= 0.00814(8), U_{33}= 0.00721(8), U_{12}=U_{13}=0, U_{23}= 0.00077(6); Sm3: U_{11}= 0.00672(5), U_{22}= 0.00672(5), U_{33}= 0.00706(8), U_{12}= 0.00053(6), U_{13}= 0.00010(4), U_{23}= 0.00010(4); Sm4: U_{11}= 0.00604(8), U_{22}= 0.00776(8), U_{33}= 0.00728(8), U_{12}=U_{13}=0, U_{23}= 0.00011(5); Sm5: U_{11}= 0.00664(5), U_{22}= 0.00664(5), U_{33}= 0.00668(8), U_{12}= 0.00037(6), U_{13}= 0.00027(4), U_{23}= 0.00027(4); Sm6: U_{11}= 0.00699(6), U_{22}= 0.00722(6), U_{33}= 0.00620(6), U_{12}= 0.00074(4),

U_{13}= -0.00016(4), U_{23}= 0.00005(4); Ge3: U_{11}= 0.0067(2), U_{22}= 0.0069(2), U_{33}= 0.0078(2), U_{12}=U_{13}=0, U_{23}= 0.0005(1); Ge4: U_{11}= 0.0066(1), U_{22}= 0.0066(1), U_{33}= 0.0074(2), U_{12}= 0.0003(1), U_{13}= -0.00012(8), U_{23}= -0.00012(8); Ge5: U_{11}= 0.0068(2), U_{22}= 0.0074(2), U_{33}= 0.0077(2), U_{12}=U_{13}=0, U_{23}= 0.0007(1); Ge7: U_{11}= 0.0087(2), U_{22}= 0.0087(2), U_{33}= 0.0082(2), U_{12}= 0.0023(1), U_{13}= 0.00033(9), U_{23}= 0.00033(9); Ge8: U_{11}= 0.0066(1), U_{22}= 0.0066(1), U_{33}= 0.0081(2), U_{12}= 0.0001(1), U_{13}= 0.00019(8), U_{23}= 0.00019(8).

Table S4. Selected interatomic distances for $Sm_{26.25}Ge_{22.75}O_5$.

Atom		Distance / Å	Atom		Distance / Å
Sm1	- 4 Sm5	3.5077(2)	Ge1_1	- 4 Ge7	2.7304(5)
	- 4 Sm6	3.3723(2)		- 4 Sm4	3.388(3)
	- 2 Ge3	3.3097(4)	Sm/Ge1_2	- 4 Ge7	2.7217(4)
	- 2 Ge5	3.1694(4)		- 4 Sm2	3.505(4)
	- 2 Ge5	3.4696(5)		- 4 Sm4	3.585(4)
	- 1 O1	2.4758(3)	Ge1_3	- 1 Ge1_4	2.62(3)
	- 2 O2	2.4387(9)		- 1 Ge2‘	2.76(2)
Sm2	- 2 Sm2	3.9530(2)		- 4 Ge7	2.968(8)
	- 2 Sm3	3.7553(2)		- 4 Sm4	2.928(7)
	- 2 Sm5	3.6779(2)	Ge1_4	- 1 Ge1_4	2.62(3)
	- 2 Sm6	3.5587(2)		- 1 Ge6	2.50(2)
	- 1 Sm/Ge1_2	3.505(4)		- 4 Ge7	3.08(1)
	- 1 Ge1_4	2.898(6)	Ge2	- 1 Ge6	3.0(1)
	- 2 Ge4	3.1882(3)		- 4 Sm3	3.2416(1)
	- 1 Ge6	3.292(1)		- 4 Sm4	3.060(5)
	- 2 Ge7	3.0495(4)	Ge2‘	- 1 Ge1_3	2.76(2)
	- 1 O2	2.400(3)		- 1 Ge6	2.644(4)
Sm3	- 2 Sm2	3.7553(2)		- 4 Sm3	3.242(1)
	- 2 Sm3	4.1726(2)		- 4 Sm4	3.231(2)
	- 2 Sm4	3.7617(2)	Ge3	- 1 Sm1	3.3097(4)
	- 2 Sm5	4.0386(2)		- 2 Sm3	2.9978(3)
	- 2 Sm6	3.8380(2)		- 1 Sm4	3.1877(4)
	- 1 Ge2	3.2416(1)		- 2 Sm5	3.0242(3)
	- 2 Ge3	2.9978(3)		- 2 Sm6	3.2078(3)
	- 1 Ge4	3.1540(4)	Ge4	- 1 Ge4	2.7103(5)
	- 1 Ge4	3.2437(3)		- 1 Ge7	3.0254(5)
	- 1 Ge6	3.2248(9)		- 2 Sm2	3.1882(3)
	- 1 Ge8	3.3387(4)		- 2 Sm3	3.1540(4)
Sm4	- 2 Sm3	3.7617(2)		- 2 Sm5	3.1222(3)
	- 2 Sm4	3.8678(2)		- 2 Sm6	3.1383(4)
	- 2 Sm6	3.8038(2)	Ge5	- 2 Ge5	2.5610(4)
	- 2 Sm6	4.0185(2)		- 1 Sm1	3.1694(4)
	- 1 Ge1_1	3.388(3)		- 1 Sm1	3.4696(5)
	- 1 Sm/Ge1_2	3.585(4)		- 1 Sm4	2.9817(5)
	- 1 Ge1_3	2.928(7)		- 2 Sm6	2.9691(3)
	- 1 Ge2	3.060(5)		- 2 Sm6	3.0887(3)
	- 1 Ge3	3.1877(4)	Ge6	- 1 Ge1_4	2.50(2)
	- 1 Ge5	2.9817(5)		- 1 Ge2’	2.644(4)
	- 2 Ge7	3.0565(4)		- 4 Sm2	3.292(1)
	- 2 Ge8	2.9276(3)		- 4 Sm3	3.2248(9)
Sm5	- 2 Sm1	3.5077(2)	Ge6’	- 1 Ge2’	3.03(1)
	- 2 Sm2	3.6779(2)		- 4 Sm2	3.106(4)
	- 2 Sm3	4.0386(2)		- 4 Sm3	3.398(5)
	- 2 Sm5	3.5140(2)	Ge7	- 1 Ge1_1	2.7304(5)
	- 2 Sm6	3.4863(2)		- 1 Sm/Ge1_2	2.7218(4)
	- 2 Ge3	3.0242(3)		- 1 Ge1_3	2.968(8)
	- 2 Ge4	3.1222(3)		- 1 Ge1_4	3.08(1)
	- 1 O1	2.4848(1)		- 1 Ge4	3.0254(5)
	- 2 O2	2.568(2)		- 1 Ge8	2.5894(5)
Sm6	- 1 Sm1	3.3723(2)		- 2 Sm2	3.0495(4)
	- 1 Sm2	3.5587(2)		- 2 Sm4	3.0565(4)
	- 1 Sm3	3.8380(2)		- 2 Sm6	3.0061(4)
	- 1 Sm4	3.8038(2)	Ge8	- 1 Ge7	2.5894(5)
	- 1 Sm4	4.0185(2)		- 1 Ge8	2.4970(4)
	- 1 Sm5	3.4863(2)		- 1 Sm3	3.3387(4)
	- 1 Sm6	3.8569(2)		- 2 Sm4	2.9276(3)
	- 1 Sm6	3.9798(2)		- 2 Sm6	2.9767(4)
	- 1 Ge3	3.2078(3)		- 2 Sm6	3.2107(4)
	- 1 Ge4	3.1383(4)	O1	- 2 Sm1	2.4758(3)
	- 1 Ge5	2.9691(3)		- 4 Sm5	2.4848(1)
	- 1 Ge5	3.0887(3)	O2	- 1 Sm1	2.4387(9)
	- 1 Ge7	3.0061(4)		- 1 Sm2	2.400(3)
	- 1 Ge8	2.9767(4)		- 2 Sm5	2.568(2)
	- 1 Ge8	3.2107(4)		- 2 Sm6	2.565(3)
	- 1 O2	2.565(3)			

4. Structural motifs in related compounds

Table S5. Structural motifs in selected suboxides.

Compound	Coordination polyhedra around O	Coordination polyhedra for non-oxygen atoms	Ref.
TiO	O in octahedral interstices of *fcc* Ti lattice	Vacancy-ordered Ti_4 tetrahedra, edge-sharing Ti octahedra	2
NbO	O in octahedral sites of *fcc* Nb lattice	Nb_4 tetrahedra and Nb_6 octahedra with short Nb–Nb bonds	3
MoO_2	O in distorted octahedra	Chains of edge-sharing MoO_6 octahedra, short Mo–Mo bonds	4
Magnéli phases (Ti_4O_7, Ti_5O_9)	O in octahedra, vacancies ordered along shear planes	Chains of TiO_6 octahedra separated by crystallographic shear planes	5
Cs_7O	$Cs_{11}O_3$-clusters, planarly stacked in columns		6
Cs_4O	≙ $[Cs_{11}O_3]Cs$, stacking of tilted $Cs_{11}O_3$-clusters with Cs in octahedral voids		7
$Cs_{11}O_3$	stacking of tilted $Cs_{11}O_3$-clusters		8
Rb_9O_2	face-sharing trigonal prisms form Rb_9O_2-clusters, distorted dense packed Rb lattice		9
Cs_9InO_4	$[InO_4]^{5-}$ tetrahedra	Cs_{12} cuboctahedra (face-sharing into columns)	10
A_9MO_9 (*A* = Rb, Cs; *M* = Al, Ga, In, Fe, Sc)	Isolated $[MO_4]$ tetrahedra	Tetrahedra encapsulated in A_{12} polyhedra	11
Cs_8Tl_8O	$[OCs_{6+2}]^{6+}$	$[Tl_8]^{6-}$ deltahedra	12
$A_{10}Tl_6O_2$ (*A* = K, Rb)	O in A_6 octahedra	$[Tl_6]^{6-}$ compressed octahedra (hypoelectronic clusters)	13
$Sr_{21}Si_2O_5C_6$	O in distorted La_6 octahedra, arranged in $[O_5Sm_{18}]$-cluster	$[SiSr_{12}]$ icosahedra, distorted $[CSr_6]$ octahedra, other complex polyhedra	14
$La_{26}Ge_{19}M_5O_5$ (*M* = Ag, Cu)	O in distorted La_6 octahedra, arranged in $[O_5Sm_{18}]$-cluster	Ge polyanionic framework, isolated Ge-butterfly anions (Zintl-like) + La–*M* cluster units	1

5. Electronic structure and chemical bonding

The bonding picture of the principal structural building blocks, except for Sm octahedra, for the hypothetical oxygen-free $Sm_{26.5}Ge_{22.5}$ compound is roughly the same (Tables S6 and S7, and Figure 4). In the absence of oxygen anions inside the Sm octahedra, a strongly localized ELF (Figure 4) is observed instead and reflects the multicenter Sm-Sm bonding. In the band structure of $Sm_{26.5}Ge_{22.5}$ (Figure S13), where the Sm and O flat bands were previously located, a band gap is now evident. Instead, stronger Sm-Sm interactions within the central Sm_6-octahedra appear in the proximity to the Fermi level, which is clearly visible in the *l-m* decomposed band structure (Figure S13). The Sm d_{xy} and d_{z2} states have weak but clearly pronounced bonding character, and the IpCOBI for 2-center Sm1-Sm1 bonding is 0.095, Sm5-Sm5 (along the diagonal) is 0.081, and for the 3-center Sm1-Sm5-Sm1 and Sm5-Sm1-Sm5 are 0.033 and 0.031, respectively. The topological analysis of the electron density revealed a non-nuclear attractor at the center of the Sm_6-octahedra with a charge of 0.183 e^-.

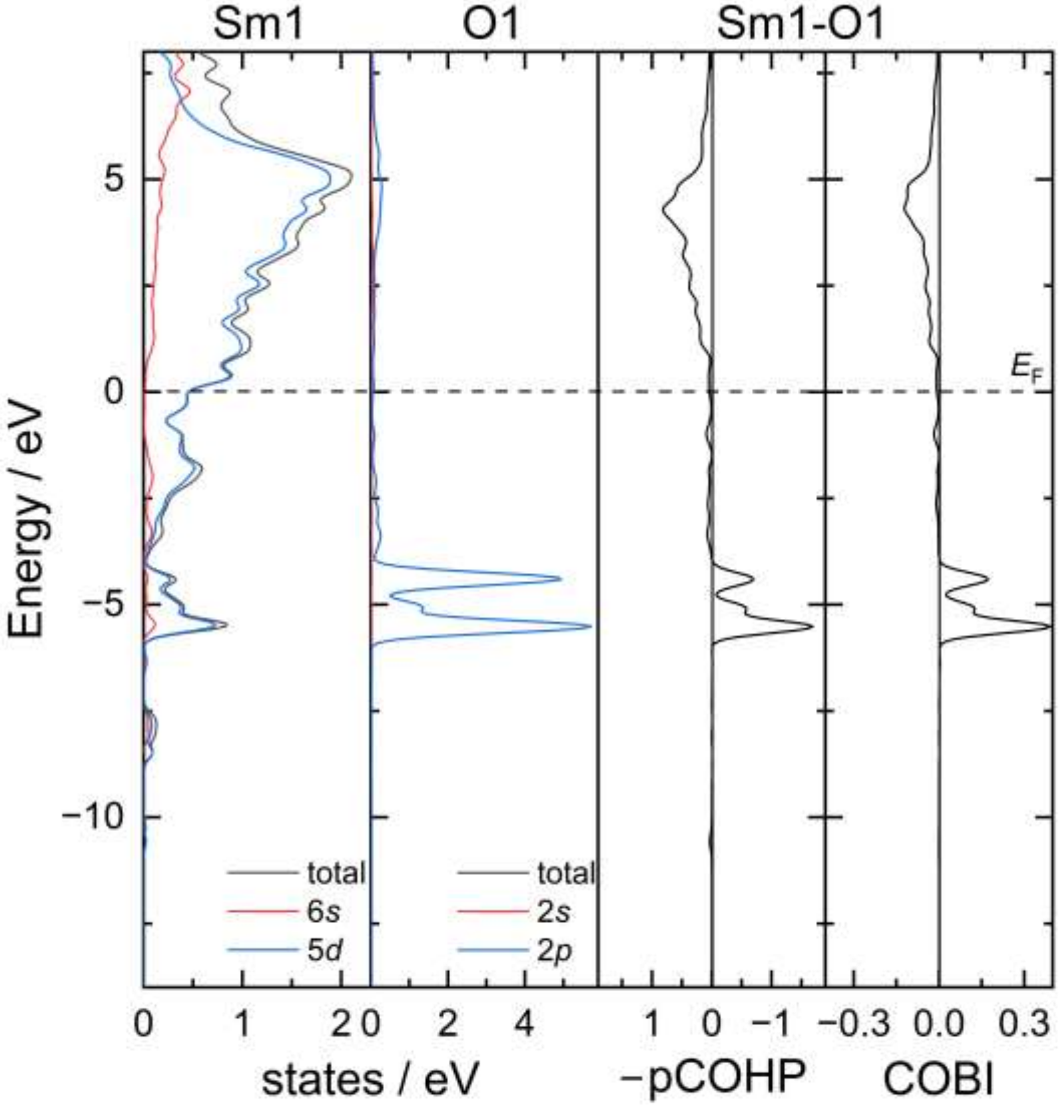

Figure S7. DOS, COHP, and COBI chemical bonding analysis of Sm1 and O1 as part of the O@Sm_6 octahedra.

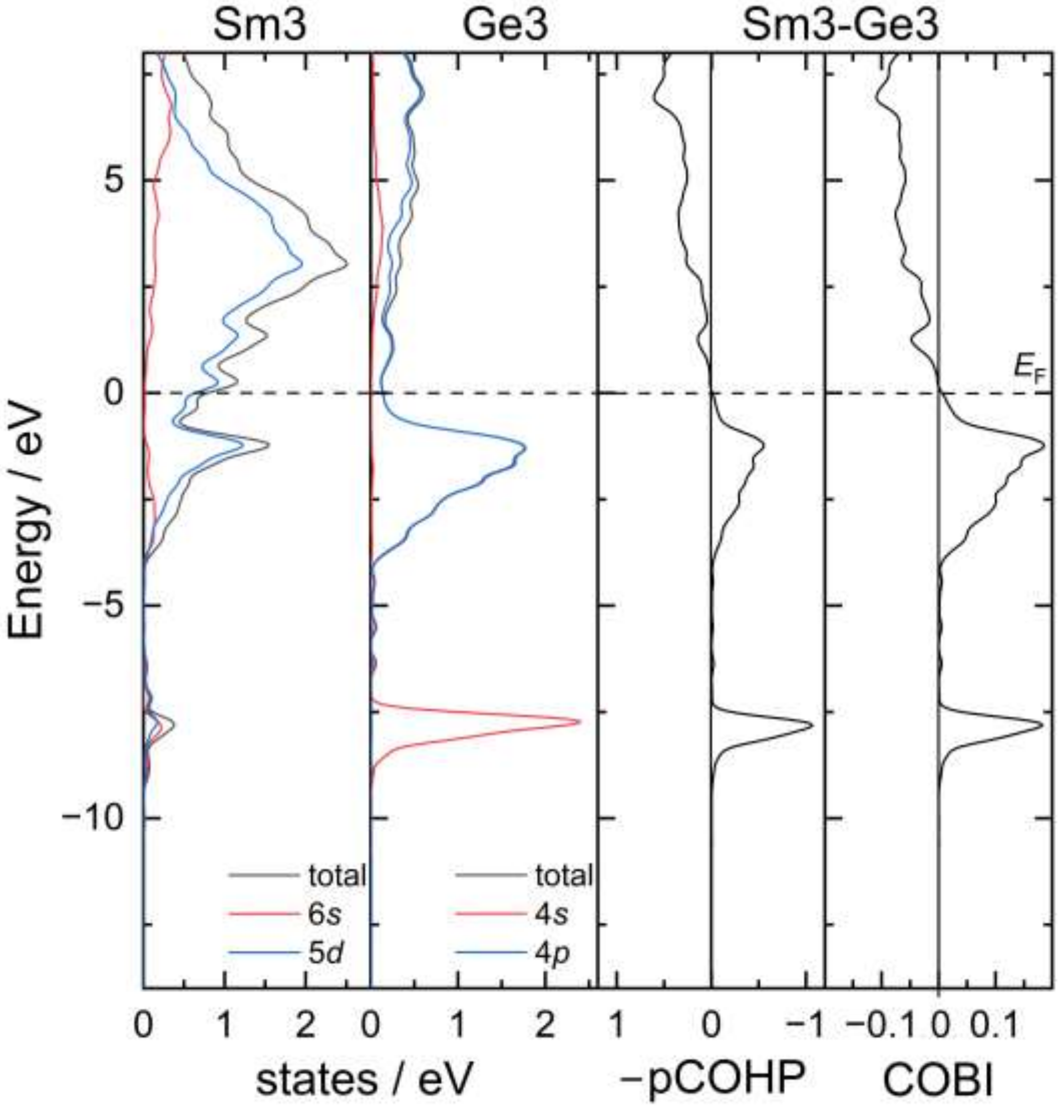

Figure S8. DOS, COHP, and COBI chemical bonding analysis of Sm3 and Ge3 as part of the trigonal prisms formed by Sm surrounding Ge.

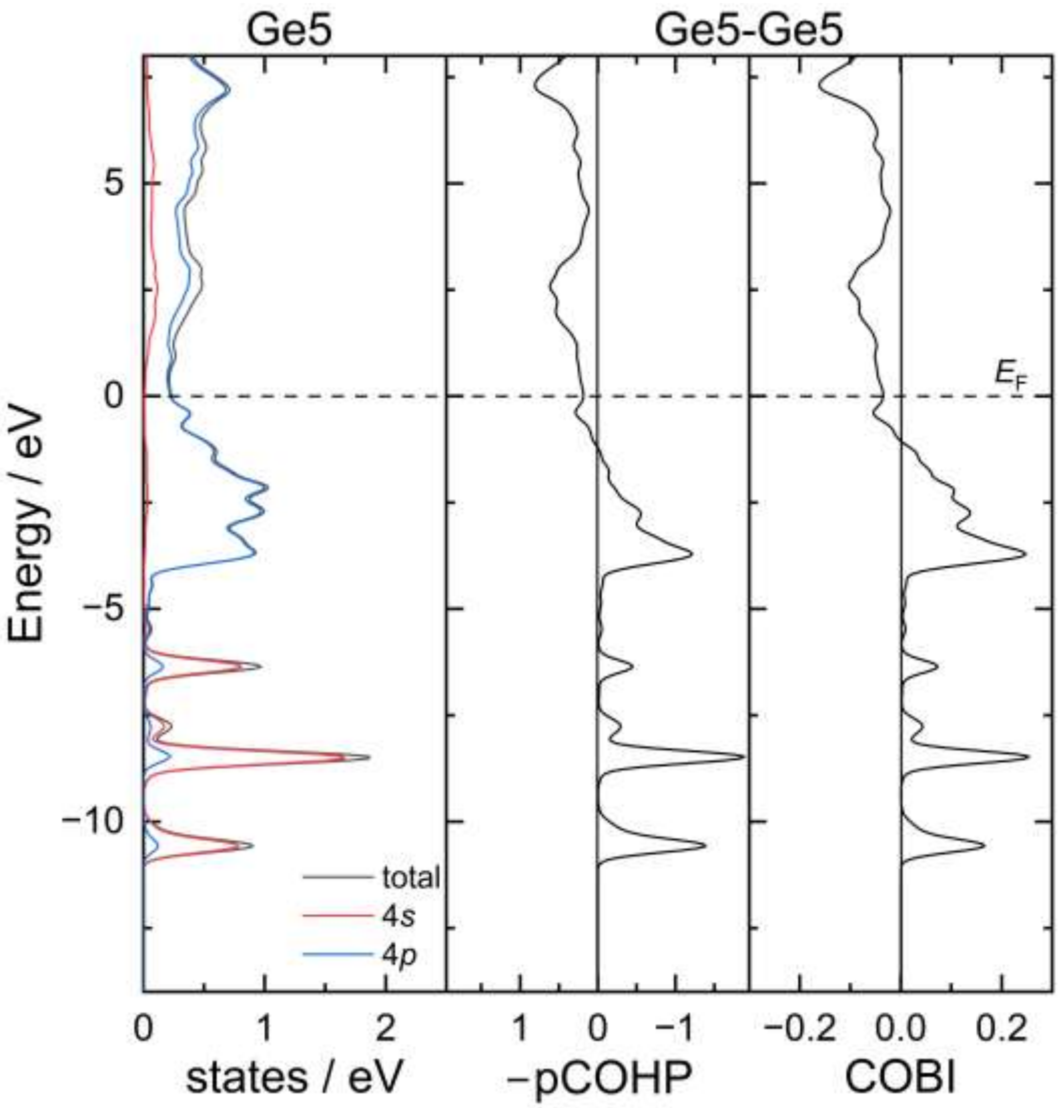

Figure S9. DOS, COHP, and COBI chemical bonding analysis of Ge5 forming butterfly units.

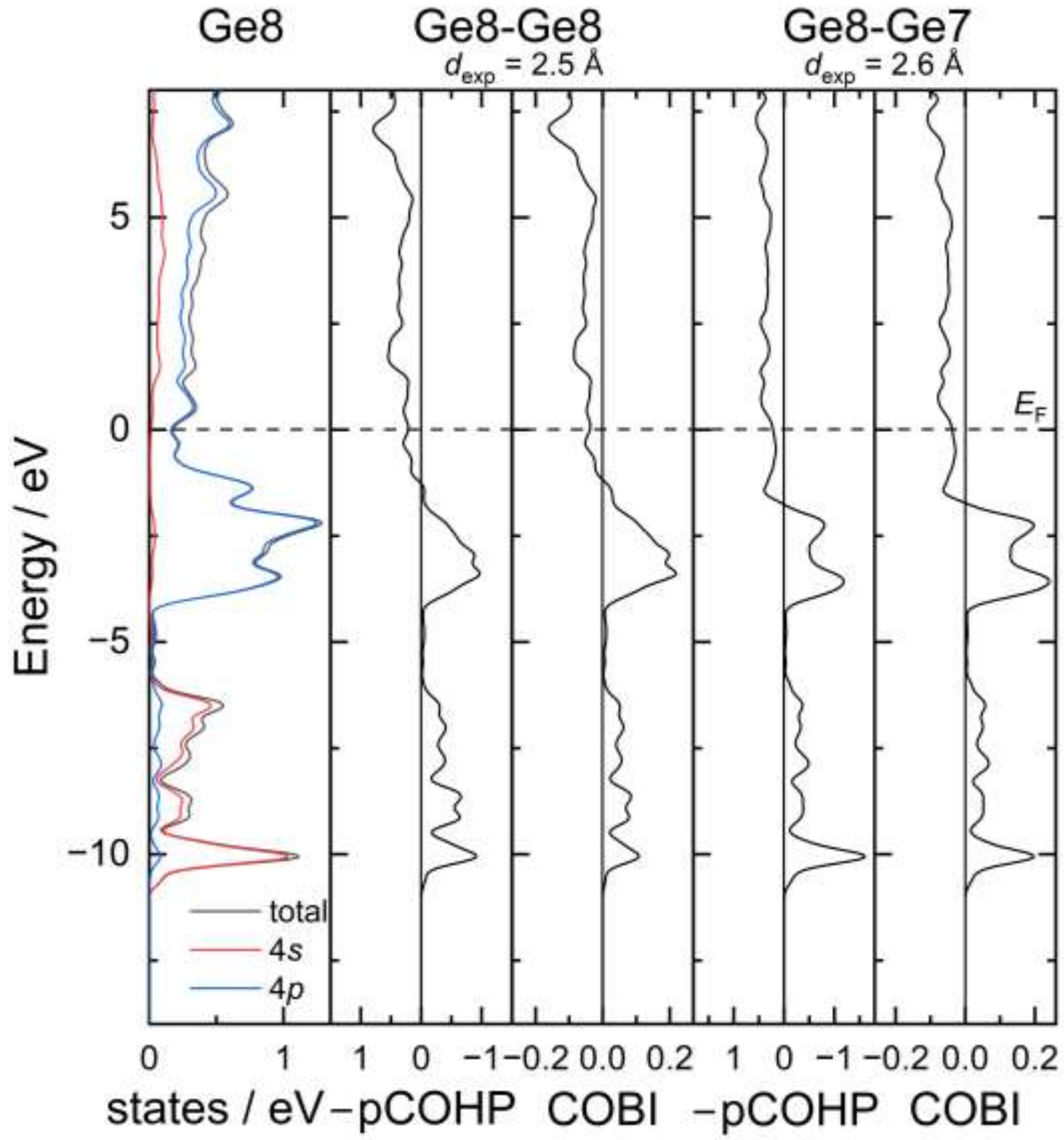

Figure S10. DOS, COHP, and COBI chemical bonding analysis of Ge8 and Ge7 comprising short Ge-Ge bonds within the Ge-subnetwork.

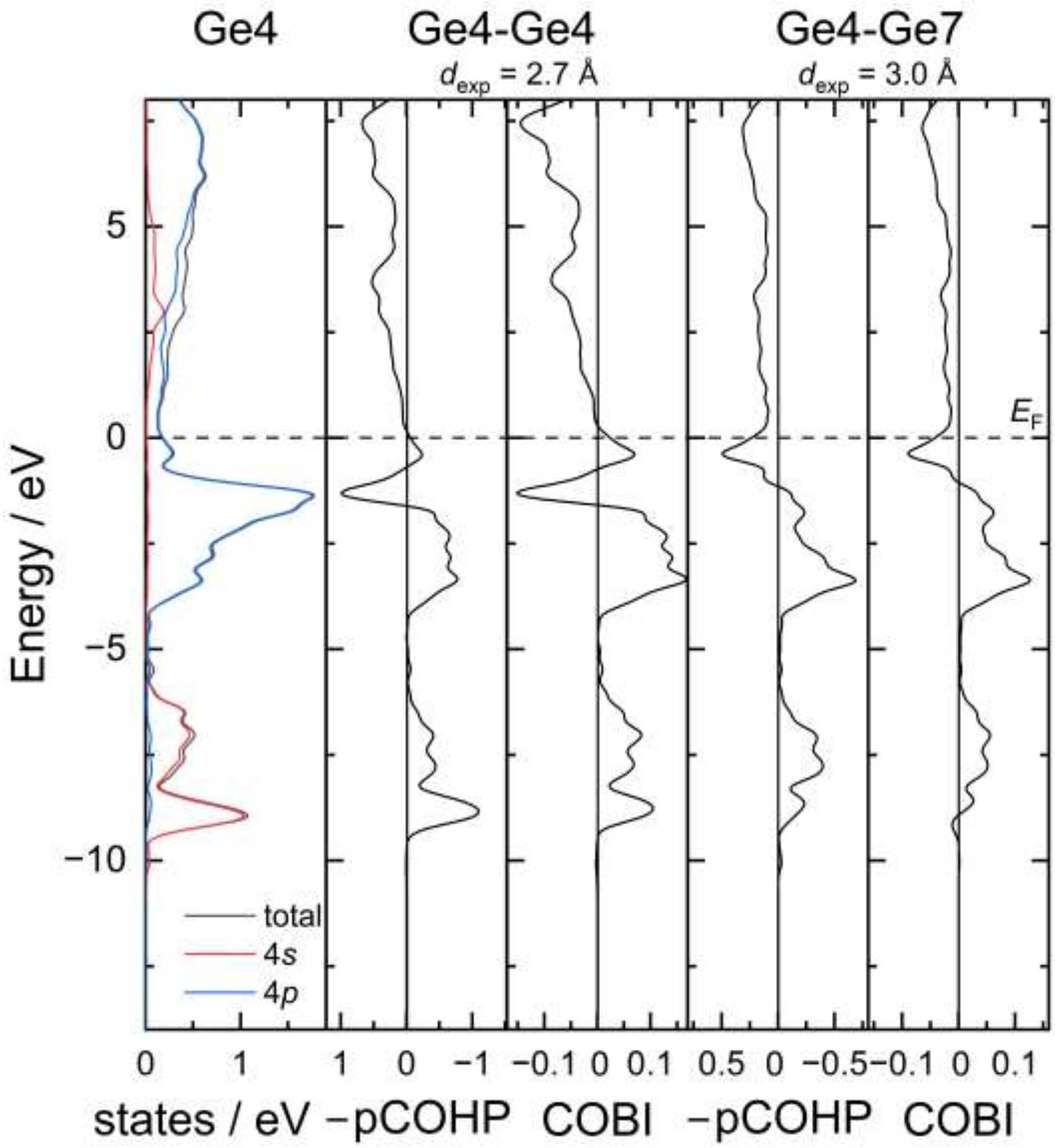

Figure S11. DOS, COHP, and COBI chemical bonding analysis of Ge4 and Ge7 comprising elongated Ge-Ge bonds within the Ge-subnetwork.

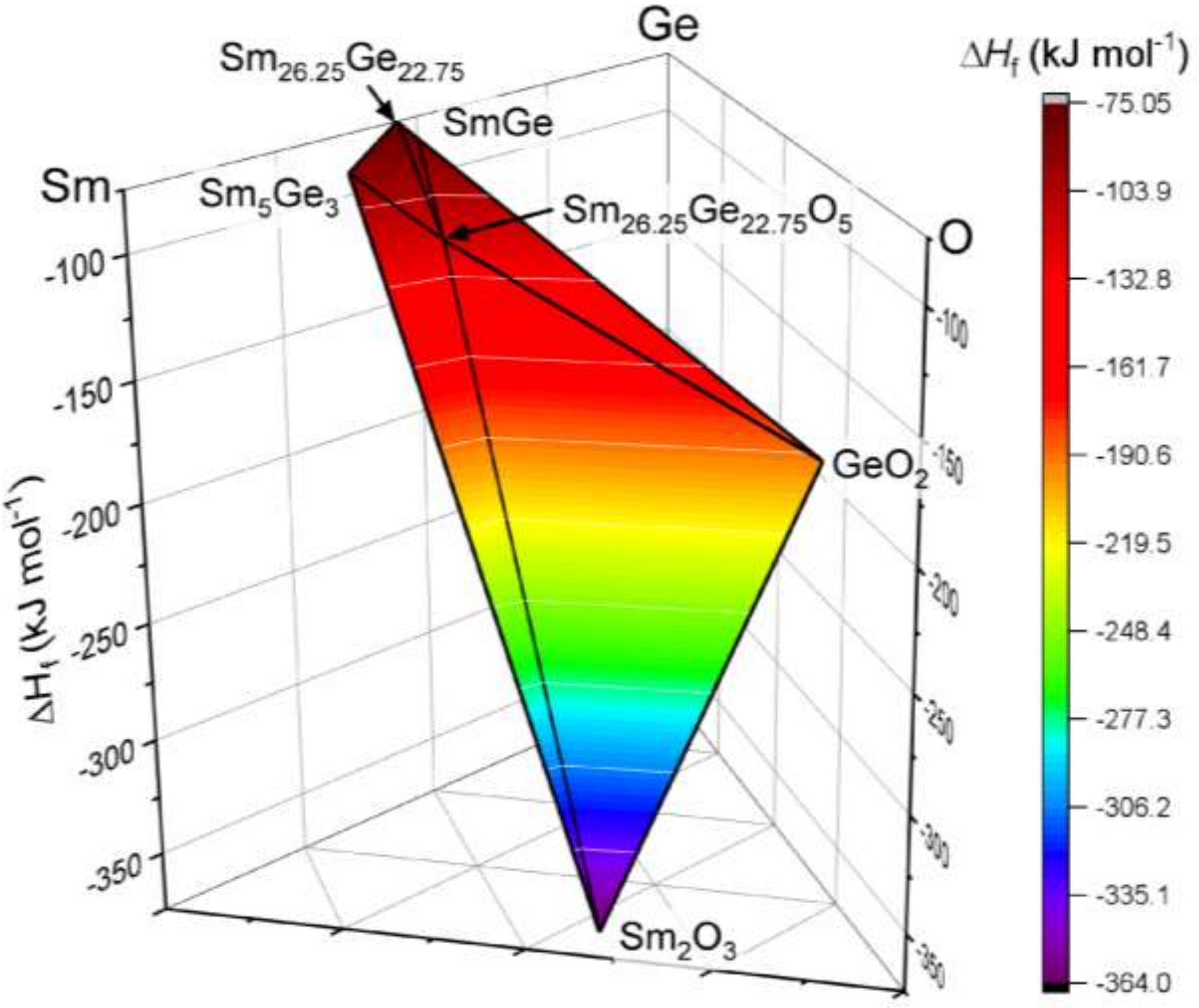

Figure S12. Enthalpies of formation of the $Sm_{26.5}Ge_{22.5}O_5$ suboxide, binary compounds around the hypothetical $Sm_{26.5}Ge_{22.5}$, and binary GeO_2 and Sm_2O_3 oxides.[15]

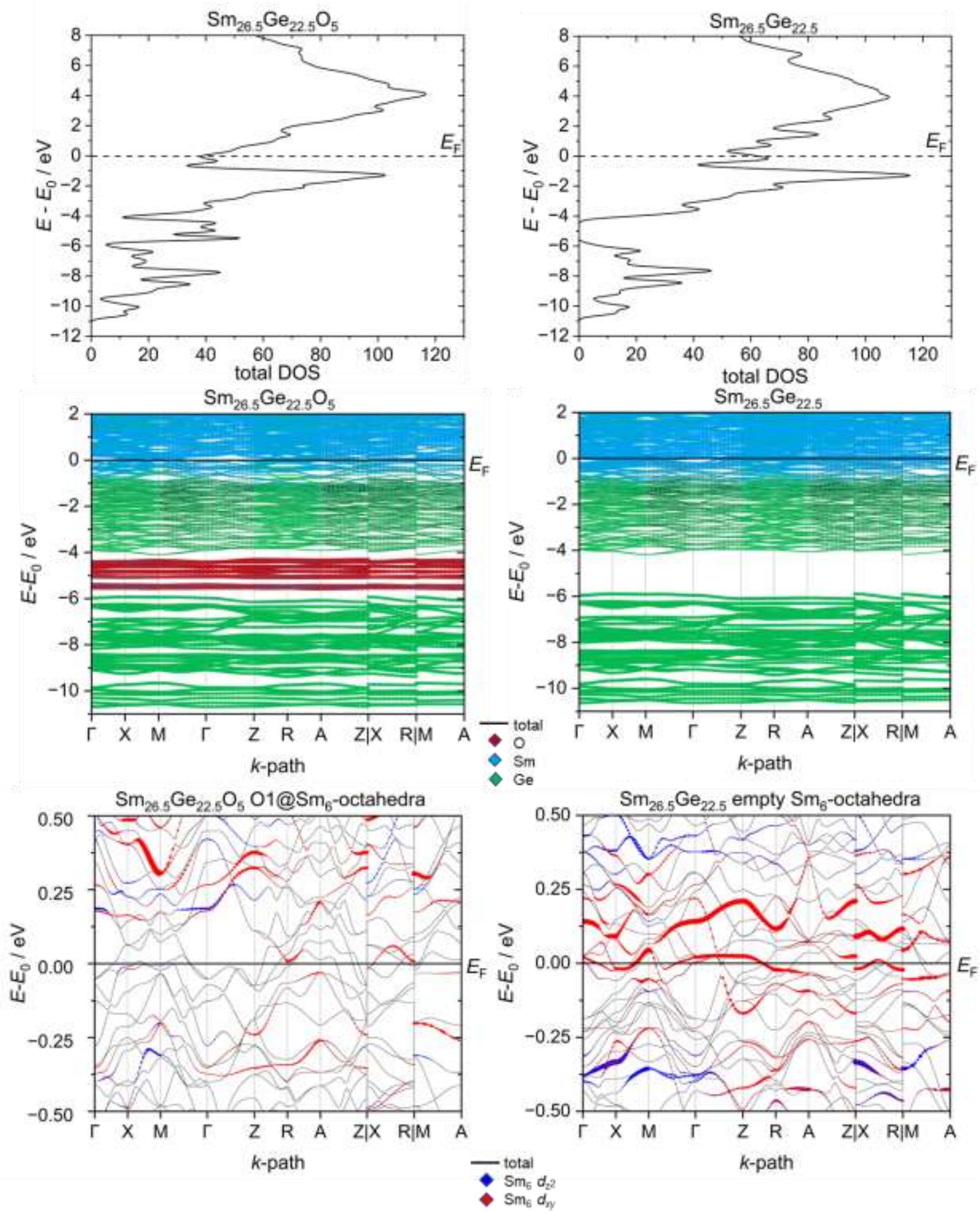

Figure S13. Total DOS (top), electronic band structure (middle) and band structure for the Sm_6 octahedra (bottom) for $Sm_{26.5}Ge_{22.5}O_5$ (left panel) and $Sm_{26.5}Ge_{22.5}$.

Table S6. Atomic charges in $Sm_{26.5}Ge_{22.5}O_5$ and the hypothetical compound $Sm_{26.5}Ge_{22.5}$.

Atom	$Sm_{26.5}Ge_{22.5}O_5$			$Sm_{26.5}Ge_{22.5}$		
	Bader	Mulliken	Löwdin	Bader	Mulliken	Löwdin
Sm1 (4*f*)	+1.445	+0.90	+0.57	+0.568	+0.37	+0.26
Sm2 (8*i*)	+1.351	+0.73	+0.44	+1.159	+0.58	+0.36
Sm3 (8*j*)	+1.309	+0.68	+0.41	+1.305	+0.70	+0.43
Sm4 (8*i*)	+1.249	+0.60	+0.23	+1.259	+0.62	+0.26
Sm5 (8*h*)	+1.477	+0.83	+0.54	+0.828	+0.36	+0.22
Sm6 (16*k*)	+1.276	+0.62	+0.27	+1.121	+0.51	+0.18
Sm_{Ge} (2*c*)	+1.050	+0.34	+0.14	+1.050	+0.38	+0.15
Ge_{Sm} (2*c*)	-0.442	-0.30	-0.10	-0.454	-0.32	-0.13
Ge2 (2*c*)	-1.521	-0.76	-0.48	-1.533	-0.78	-0.51
Ge3 (8*i*)	-1.710	-0.83	-0.49	-1.788	-0.85	-0.55
Ge4 (8*j*)	-1.342	-0.69	-0.37	-1.352	-0.65	-0.36
Ge5 (8*i*)	-1.007	-0.49	-0.17	-1.032	-0.49	-0.18
Ge6 (2*c*)	-1.589	-0.81	-0.53	-1.626	-0.81	-0.53
Ge7 (8*j*)	-1.147	-0.56	-0.24	-1.134	-0.57	-0.25
Ge8 (8*j*)	-1.052	-0.51	-0.16	-1.054	-0.50	-0.16
O1 (2*b*)	-1.306	-0.85	-0.61	-	-	-
O2 (8*i*)	-1.376	-0.83	-0.60	-	-	-

Table S7. Interatomic distances (in Å), ICOHP (in eV), and ICOBI for the atomic pairs in optimized structures of $Sm_{26.5}Ge_{22.5}O_5$ and the hypothetical compound $Sm_{26.5}Ge_{22.5}$.

Atoms	$Sm_{26.5}Ge_{22.5}O_5$			$Sm_{26.5}Ge_{22.5}$		
	distance	-ICOHP	ICOBI	distance	-ICOHP	ICOBI
**Oxygen polyhedra**						
O1-Sm1	2.51165	2.14821	0.32619			
	2.48756	2.26381	0.34239			
O1-Sm5	2.51751	2.15560	0.32681			
O2-Sm1	2.45633	2.54284	0.39685			
O2-Sm2	2.46259	2.54144	0.41696			
O2-Sm5	2.56455	2.03096	0.32288			
O2-Sm6	2.62677	1.78122	0.28445			
**Ge@Sm polyhedra**						
Ge3-Sm1	3.33910	1.23572	0.27158	3.38860	1.18287	0.27013
Ge3-Sm3	3.03431	2.27254	0.47167	3.06788	2.16281	0.46364
Ge3-Sm4	3.20423	1.61218	0.32621	3.27000	1.46966	0.31215
Ge3-Sm5	3.04265	2.11447	0.42970	3.05439	2.11698	0.43761
Ge3-Sm6	3.25200	1.44328	0.30594	3.22813	1.55949	0.33375
**Ge-butterflies**						
Ge5-Ge5	2.59856	3.10605	0.55745	2.60273	3.11744	0.56666
**Ge-chains (d < 2.6 Å)**						
Ge8-Ge8	2.57824	3.22249	0.56247	2.55861	3.35516	0.58955
Ge7-Ge8	2.55098	3.37921	0.58483	2.57647	3.17827	0.55145
**Ge-network (2.6 - 2.8 Å)**						
Ge4-Ge4	2.74014	2.55635	0.45697	2.89904	1.83435	0.33229
Ge1_1-Ge7	2.74301	2.48872	0.45634	2.80021	2.21684	0.41594
Ge7-Ge8	2.64183	2.87277	0.51499	2.60142	3.08882	0.54593
Ge2-Ge6	2.69335	3.05713	0.63323	2.71102	2.90168	0.61169
**Ge-network (2.8 - 3.1 Å)**						
Ge4-Ge7	2.96815	1.42370	0.24953	2.88476	1.76614	0.32249
	3.01627	1.29893	0.22714	2.92774	1.62430	0.29425

Table S8. Components of the stiffness tensor c_{ij} (GPa) for the $Sm_{26.5}Ge_{22.5}O_5$ suboxide and hypothetical $Sm_{26.5}Ge_{22.5}$ compound.

Compound	c_{11}	c_{12}	c_{13}	c_{33}	c_{44}	c_{66}
$Sm_{26.5}Ge_{22.5}O_5$	164.631	40.687	45.957	127.786	50.049	48.529
$Sm_{26.5}Ge_{22.5}$	132.173	33.496	40.540	97.425	39.172	39.531

Table S9. Calculated elastic properties (Hill notation) of $Sm_{26.5}Ge_{22.5}O_5$ and a hypothetical $Sm_{26.5}Ge_{22.5}$ compound: bulk modulus *B* (GPa), Young's modulus *E* (GPa), shear modulus *G* (GPa), Poisson's ratio *v*, Pugh's ratio *B*/*G*, Vickers hardness *VH* from Chen's[16] and Tian's[17] models (GPa), Cauchy pressure P_c (GPa), Debye temperature θ_D (K).

Compound	*B*	*E*	*G*	*v*	*B*/*G*	*VH* (Chen)	*VH* (Tian)	P_c	θ_D
$Sm_{26.5}Ge_{22.5}O_5$	79.850	126.222	51.038	0.237	1.565	8.751	8.953	-9.4	299.1
$Sm_{26.5}Ge_{22.5}$	65.228	98.951	39.670	0.247	1.644	6.574	7.079	-5.7	258.4

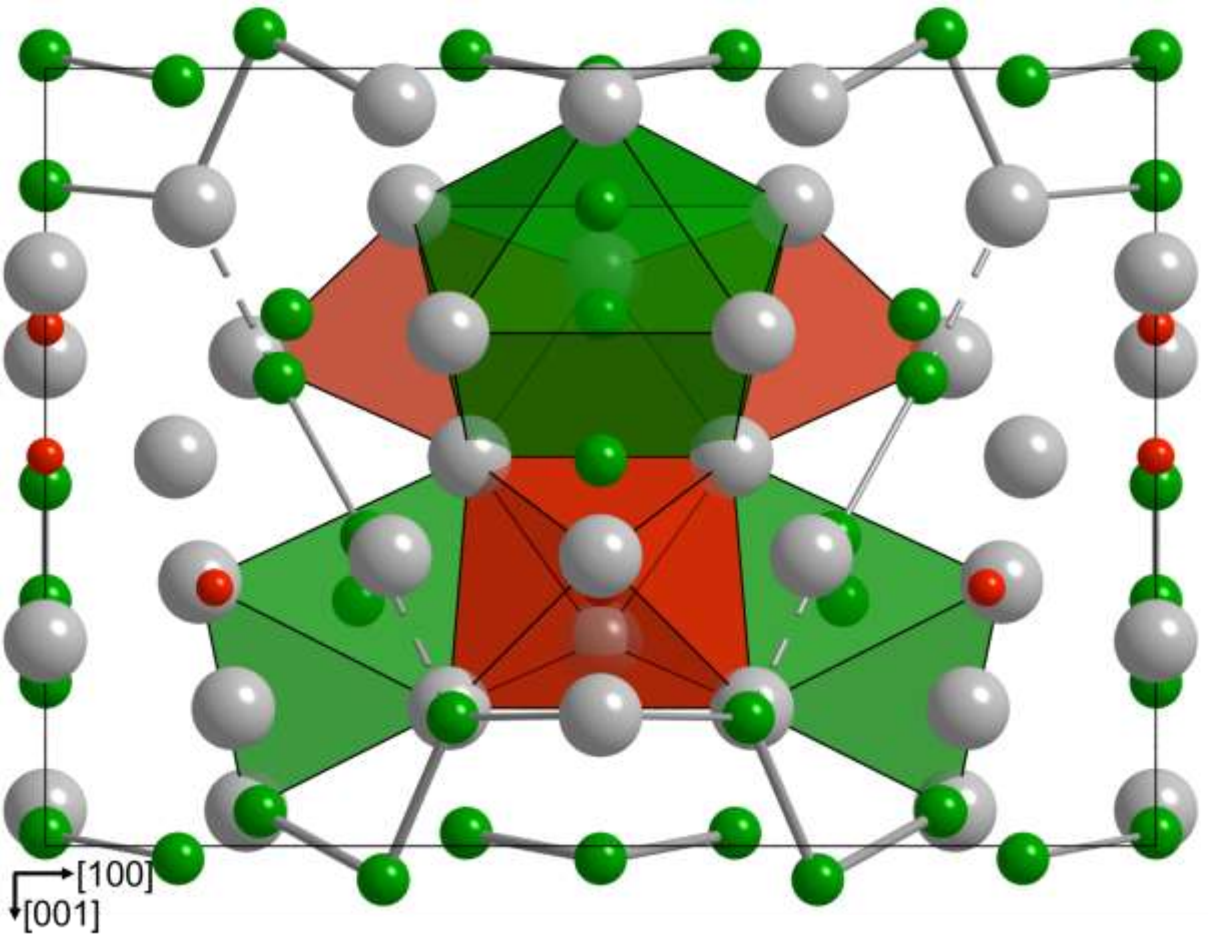

Figure S14. Crystal structure of $Sm_{26.5}Ge_{22.5}O_5$ (gray: Sm, green: Ge, red: O) optimized in space group *P*1.

References

(1) Ma, X.; Wang, L.; Deng, L.; Gooch, M.; Tang, Z.; Chu, C.-W.; Guloy, A. M. $La_{26}Ge_{19}M_5O_5$ (*M* = Ag, Cu): Rare-Earth Metal Suboxide Superconductors with $La_{18}O_5$ Cluster Units. *J. Am. Chem. Soc.* **2023**, *145* (1), 12–16. DOI: 10.1021/jacs.2c11077.
(2) Watanabé, D.; Castles, J. R.; Jostsons, A.; Malin, A. S. The ordered structure of TiO. *Acta Cryst* **1967**, *23* (2), 307–313. DOI: 10.1107/S0365110X67002634.
(3) Burdett, J. K.; Mitchell, J. F. Pair potentials and the ordered defect structure of niobium monoxide. *Inorg. Chem.* **1993**, *32* (23), 5004–5006. DOI: 10.1021/ic00075a010.
(4) Brandt, B. G.; Skapski, A. C.; Thom, E.; Stoll, E.; Eriksson, G.; Blinc, R.; Paušak, S.; Ehrenberg, L.; Dumanović, J. A Refinement of the Crystal Structure of Molybdenum Dioxide. *Acta Chem. Scand.* **1967**, *21*, 661–672. DOI: 10.3891/acta.chem.scand.21-0661.
(5) Malik, H.; Sarkar, S.; Mohanty, S.; Carlson, K. Modelling and synthesis of Magnéli Phases in ordered titanium oxide nanotubes with preserved morphology. *Sci. Rep.* **2020**, *10* (1), 8050. DOI: 10.1038/s41598-020-64918-0.
(6) Simon, A. Über Alkalimetall-Suboxide. VII. Das metallreichste Cäsiumoxid—Cs_7O. *Z. Anorg. Allg. Chem.* **1976**, *422* (3), 208–218. DOI: 10.1002/zaac.19764220303.
(7) Simon, A.; Deiseroth, H.-J.; Westerbeck, E.; Hillenkötter, B. Über Alkalimetallsuboxide. VIII. Untersuchungen zum Aufbau von Tetracäsiumoxid. *Z. Anorg. Allg. Chem.* **1976**, *423* (3), 203–211. DOI: 10.1002/zaac.19764230303.
(8) Simon, A.; Westerbeck, E. Über Suboxide der Alkalimetalle. 10. Das „komplexe Metall" $Cs_{11}O_3$. *Z. Anorg. Allg. Chem.* **1977**, *428* (1), 187–198. DOI: 10.1002/zaac.19774280123.
(9) Simon, A. Über Alkalimetall Suboxide. 11. Das „komplexe Metall" Rb_9O_2. *Z. Anorg. Allg. Chem.* **1977**, *431* (1), 5–16. DOI: 10.1002/zaac.19774310101.
(10) Hoch, C.; Bender, J.; Simon, A. Suboxides with complex anions: the suboxoindate Cs_9InO_4. *Angew. Chem. Int. Ed.* **2009**, *48* (13), 2415–2417. DOI: 10.1002/anie.200805736.
(11) Hoch, C.; Bender, J.; Wohlfarth, A.; Simon, A. Die Suboxometallate A_9MO_4 (*A* = Rb, Cs; *M* = Al, Ga, In, Fe, Sc). *Z. Anorg. Allg. Chem.* **2009**, *635* (12), 1777–1782. DOI: 10.1002/zaac.200900193.
(12) Karpov, A.; Jansen, M. Tl_8^{6-} in Cs_8Tl_8O: a naked eight-vertex closo-deltaeder as a cluster anion. *Angew. Chem. Int. Ed.* **2005**, *44* (46), 7639–7643. DOI: 10.1002/anie.200502283.
(13) Karpov, A.; Jansen, M. $A_{10}Tl_6O_2$ (*A* = K, Rb) cluster compounds combining structural features of thallium cluster anions and of alkali metal sub-oxides. *Chem. Commun.* **2006** (16), 1706–1708. DOI: 10.1039/b601802e.
(14) Knoth, M.; Rößler, U.; Eisenmann, B. Zur Stabilisierung von Suboxidphasen durch Carbidionen: Die Kristallstruktur von $Sr_{21}Si_2O_5C_6$. *Z. Anorg. Allg. Chem.* **2005**, *631* (6-7), 1237–1240. DOI: 10.1002/zaac.200500023.
(15) Haynes, W. M., Ed. *CRC handbook of chemistry and physics*: *A ready-reference book of chemical and physical data*, 97th edition; CRC Press, 2017.
(16) Chen, X.-Q.; Niu, H.; Li, D.; Li, Y. Modeling hardness of polycrystalline materials and bulk metallic glasses. *Intermetallics* **2011**, *19* (9), 1275–1281. DOI: 10.1016/j.intermet.2011.03.026.
(17) Tian, Y.; Xu, B.; Zhao, Z. Microscopic theory of hardness and design of novel superhard crystals. *Int. J. of Refract. Met. Hard Mater.* **2012**, *33*, 93–106. DOI: 10.1016/j.ijrmhm.2012.02.021.
